# Impact of the SnRK1 protein kinase on sucrose homeostasis and the transcriptome during the diel cycle

**DOI:** 10.1093/plphys/kiab350

**Published:** 2021-07-28

**Authors:** Bruno Peixoto, Thiago A Moraes, Virginie Mengin, Leonor Margalha, Rubén Vicente, Regina Feil, Melanie Höhne, António G G Sousa, Jingtao Lilue, Mark Stitt, John E Lunn, Elena Baena-González

**Affiliations:** 1 Instituto Gulbenkian de Ciência, 2780-156 Oeiras, Portugal and GREEN-IT Bioresources for Sustainability, ITQB NOVA, 2780-157 Oeiras, Portugal; 2 Max Planck Institute of Molecular Plant Physiology, 14476 Potsdam-Golm, Germany; 3 GREEN-IT Bioresources for Sustainability, ITQB NOVA, 2780-157 Oeiras, Portugal; 4 Instituto Gulbenkian de Ciência, Bioinformatics Unit, 2780-156 Oeiras, Portugal

## Abstract

SNF1-related Kinase 1 (SnRK1) is an evolutionarily conserved protein kinase with key functions in energy management during stress responses in plants. To address a potential role of SnRK1 under favorable conditions, we performed a metabolomic and transcriptomic characterization of rosettes of 20-d-old Arabidopsis (*Arabidopsis thaliana*) plants of SnRK1 gain- and loss-of-function mutants during the regular diel cycle. Our results show that SnRK1 manipulation alters the sucrose and trehalose 6-phosphate (Tre6P) relationship, influencing how the sucrose content is translated into Tre6P accumulation and modulating the flux of carbon to the tricarboxylic acid cycle downstream of Tre6P signaling. On the other hand, daily cycles of Tre6P accumulation were accompanied by changes in SnRK1 signaling, leading to a maximum in the expression of SnRK1-induced genes at the end of the night, when Tre6P levels are lowest, and to a minimum at the end of the day, when Tre6P levels peak. The expression of SnRK1-induced genes was strongly reduced by transient Tre6P accumulation in an inducible Tre6P synthase (otsA) line, further suggesting the involvement of Tre6P in the diel oscillations in SnRK1 signaling. Transcriptional profiling of wild-type plants and SnRK1 mutants also uncovered defects that are suggestive of an iron sufficiency response and of a matching induction of sulfur acquisition and assimilation when SnRK1 is depleted. In conclusion, under favorable growth conditions, SnRK1 plays a role in sucrose homeostasis and transcriptome remodeling in autotrophic tissues and its activity is influenced by diel fluctuations in Tre6P levels.

## Teaser

Daily changes in trehalose 6-phosphate levels impact the activity of the SnRK1 protein kinase, contributing to the maintenance of sucrose homeostasis and to diel patterns of gene expression.

## Introduction

In most plants, sucrose is a major product of photosynthesis and the most common form in which carbon is exported to sink organs for growth, development and storage (Ruan, 2014). Sucrose metabolism is tightly controlled (1) to ensure that sucrose synthesis is neither too slow nor too fast to limit CO_2_ fixation and (2) to balance sucrose synthesis and export with the accumulation of carbon reserves that enable survival and growth through the night (Smith and Stitt, 2007). The inability to efficiently manage carbon resources has clear penalties on plant growth. On the one hand, incomplete starch mobilization results in yield losses due to nonproductive carbon sequestration. On the other hand, if starch is prematurely depleted during the night, the ensuing carbon starvation leads to metabolic and transcriptional responses that inhibit growth (Smith and Stitt, 2007; Stitt and Zeeman, 2012).

Although the underlying mechanisms are still poorly understood, several of the components involved in the coordinated regulation of carbon assimilation, storage and growth are known. One is trehalose 6-phosphate (Tre6P) signaling. Tre6P is a regulatory sugar that modulates primary metabolism, growth, and development. Tre6P is synthesized from glucose 6-phosphate (Glc6P) and UDP-glucose by Tre6P synthase (TPS) and is further metabolized into trehalose by Tre6P phosphatase (TPP; Lunn et al., 2014; Fichtner and Lunn, 2021). The Arabidopsis (*Arabidopsis thaliana*) genome harbors 11 genes encoding TPS or TPS-like proteins (*TPS1*–*TPS11*) and 10 genes encoding TPP proteins (*TPPA*–*TPPJ*). Class I TPS proteins (TPS1–TPS4) contain a catalytically active glucosyltransferase domain and a noncatalytic TPP-like domain. The class II proteins (TPS5–TPS11) have a similar domain structure and most of the active site residues are conserved, but they do not appear to have TPS or TPP activity (Ramon et al., 2009; Lunn et al., 2014; Delorge et al., 2015; Fichtner and Lunn, 2021).

Normally, Tre6P concentrations are very low in Arabidopsis plants (0.01–2 nmol g^−1^ FW), but their levels respond to daily sucrose peaks and also rise dramatically in response to exogenous sucrose feeding (Lunn et al., 2006; Nunes et al., 2013; Yadav et al., 2014). Indeed, a strong correlation between Tre6P and sucrose levels has been reported in many tissues and species, suggesting that Tre6P is an evolutionarily conserved proxy for the plant sucrose status (Lunn et al., 2014; Fichtner and Lunn, 2021). Most importantly, Tre6P regulates sucrose levels by promoting sucrose consumption and preventing further synthesis when Tre6P levels are high, while promoting sucrose synthesis and decreasing sucrose consumption when Tre6P levels are low (Lunn et al., 2014; Fichtner and Lunn, 2021). Using an inducible system for Tre6P production in combination with metabolomics, proteomics, and flux analyses, it was demonstrated that high Tre6P reduces sucrose production in source leaves by diverting photoassimilates into organic and amino acid synthesis (by activating nitrate reductase [NR] and phosphoenolpyruvate carboxylase [PEPC]) during the day (Figueroa et al., 2016) and by inhibiting starch degradation at night (Martins et al., 2013).

Another component of the energy management network is SNF1-Related Kinase 1 (SnRK1), an evolutionarily conserved heterotrimeric protein kinase complex composed of an α-catalytic subunit and regulatory β- and γ-subunits (SnRK1α1/α2, SnRK1β1/β2/β3, and SnRK1βγ in Arabidopsis; Broeckx et al., 2016). SnRK1 is inhibited, directly or indirectly, by sugars such as Tre6P, Glc6P, and glucose 1-phosphate (Zhang et al., 2009; Nunes et al., 2013; Zhai et al., 2018; Baena-González and Lunn, 2020), while SnRK1 signaling is activated in response to low energy levels, often associated with stress (Baena-González et al., 2007; Baena-González and Sheen, 2008; Crepin and Rolland, 2019). Upon activation, SnRK1 triggers transcriptional and metabolic responses to stimulate energy-producing catabolic processes and inhibit energy-consuming biosynthetic processes and growth (Baena-González and Sheen, 2008; Nukarinen et al., 2016), ultimately promoting stress tolerance and survival. The relevance of SnRK1 for stress responses has been validated in planta, where the overexpression of the main catalytic subunit SnRK1α1 enhances tolerance to abiotic and biotic stresses, while the opposite is true for *snrk1α* knockdown mutants (Hulsmans et al., 2016; Margalha et al., 2019).

In this study, we hypothesized that SnRK1 plays important roles in daily carbon management in the absence of any overt form of abiotic or biotic stress treatment. To investigate this, we compared wild-type plants and SnRK1 gain- and loss-of-function mutants at the metabolite and transcriptional levels at different timepoints of the day and night. Our results implicate SnRK1 in sucrose homeostasis and transcriptional regulation during normal day–night cycles in rosette leaves. They further show that this is at least partly determined by diel oscillations in Tre6P accumulation that impact SnRK1 activity.

## Results

To investigate the function of SnRK1 during the regular diel cycle, we grew plants under conditions that are only slightly limiting for growth of Arabidopsis Col-0 plants (12-h photoperiod with an irradiance of 160 µmol m^−2^ s^−1^; Sulpice et al., 2014). We used a SnRK1α1 overexpression line (*SnRK1α1-OE*; Jossier et al., 2009), and a partial SnRK1α loss-of-function mutant (*snrk1α1*^*−*^^/^^*−*^*snrk1α2*^+/^^*−*^, *sesquiα2*; Belda-Palazón et al., 2020). The *sesquiα2* line was selected as a loss-of-function mutant for two reasons. First, the molecular phenotypes of the *snrk1α1* and *snrk1α2* single mutants are mild even under stress conditions (Nukarinen et al., 2016; Belda-Palazón et al., 2020) due to functional redundancy between the two *α*-subunits (Baena-González et al., 2007). Second, despite being a stronger loss-of-function mutant than the single *snrk1α* mutants, the *sesquiα2* line does not show obvious growth phenotypes under our standard laboratory growth conditions in soil (Supplemental Figure S1A). This makes this mutant ideal for investigating a potential impact of SnRK1 on metabolism and gene expression without confounding effects derived from altered growth and development. The *SnRK1α1-OE* line was selected as a gain-of-function mutant also because it did not show obvious growth alterations under our growth conditions (Supplemental Figure S1A) and because it was generated in the same background as the *sesquiα2* mutant (Col-0; Jossier et al., 2009). Mutants and Col-0 control plants were grown for 20 d and then harvested at 4-h intervals over a period of 40 h starting at ZT8 (Supplemental Figure S1B). Using both enzymatic, as well as liquid chromatography with tandem mass spectrometry (LC–MS/MS) methodologies, we analyzed these samples for the contents of a total of 38 different metabolites related to pathways of carbon and nitrogen primary metabolism (Supplemental Table S1).

### Impact on sucrose and Tre6P

**Figure 1:**
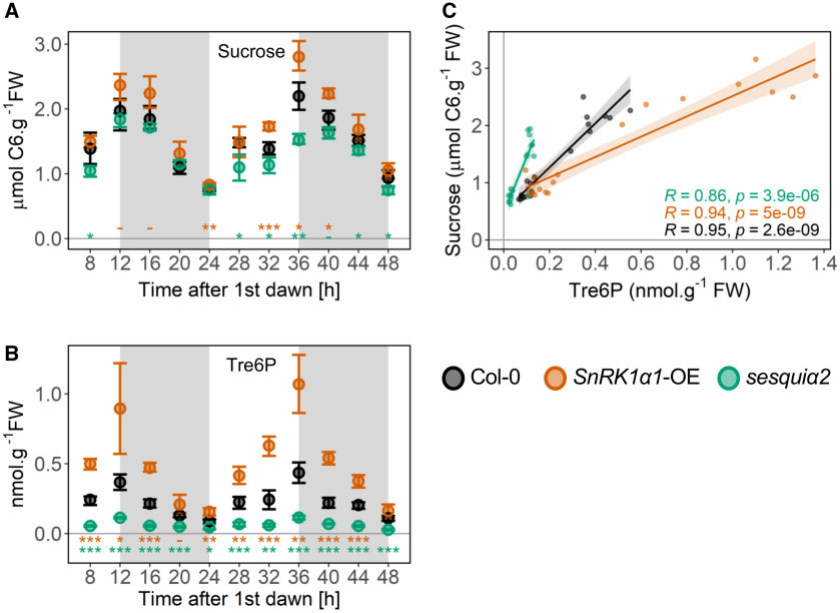
Impact of SnRK1 on sucrose and Tre6P accumulation and their relationship. Sucrose (A) and Tre6P (B) levels were quantified from 20-d-old Col-0, *SnRK1α1-OE*, and *sesquiα2* plants grown under a 12:12 photoperiod and harvested every 4 h. The night period is marked in grey. Graphs show the average of four to five biological replicates (each composed of a pool of four to five randomly sampled whole rosettes) at each time point, with error bars representing the 95% confidence interval. Asterisks denote statistically significant differences tested at each ZT for both genotypes separately (one-way ANOVA with Tukey’s post hoc test of honestly significant differences, HSD). ^-^*P* < 0.1 cases in which the Tukey’s HSD test resulted in non-significant differences; *P <* *0.05; **P <* *0.01; ***P <* *0.001. C, Impact of SnRK1 on the Tre6P-sucrose relationship. The Tre6P content of samples at ED (12 h and 36 h after first dawn) and EN (24 h and 48 h after first dawn) was plotted against the corresponding values of sucrose. *R*, Pearson correlation coefficient. Sucrose levels correspond to hexose equivalents.

As shown in Figure 1, A and B, Col-0 rosettes displayed the expected diel changes of sucrose and Tre6P, with sucrose levels peaking at the end of the day (ED) and being closely mirrored by Tre6P. The average sucrose content ranged from 0.84 to 2.08 µmol (hexose equivalents) g^−1^ FW at the end of the night (EN) and ED, respectively, while Tre6P ranged from 0.10 to 0.40 nmol g^−1^ FW at EN and ED, respectively. This resulted in Tre6P:sucrose ratios of 0.12 at EN and 0.19 at ED, consistent with values previously reported for Col-0 rosettes grown under equinoctial conditions (Yadav et al., 2014; Table 1). Qualitatively similar diel changes of sucrose and Tre6P accumulation were observed for *SnRK1α1-OE and sesquiα2* (Figure 1, A and B). However, *SnRK1α1-OE* contained higher levels of both metabolites. The differences were significant at nine time points for Tre6P (Figure 1B), and at four for sucrose (Figure 1A). Importantly, the change in Tre6P was proportionally larger than that of sucrose (2- and 1.4-fold higher at ED and EN, respectively). This led to Tre6P:sucrose ratios that were 1.9- and 1.4-fold higher than in Col-0 at ED and EN, respectively, with the main cause for this being higher Tre6P levels (Table 1, ED: 2.4-fold, EN: 1.6-fold higher). In contrast, *sesquiα2* contained lower levels of both metabolites. The differences were significant at all eleven time points for Tre6P (Figure 1B), but only at six for sucrose (Figure 1A). The change in Tre6P was again proportionally larger than that of sucrose (2.9- and 2.4-fold lower at ED and EN, respectively). This led to Tre6P:sucrose ratios that were 2.8- and 2.5-fold lower than in Col-0 at ED and EN, respectively, with the main cause for this being lower Tre6P levels (Table 1; ED: 3.5-fold, EN: 2.7-fold lower). Fructose and glucose showed very small and variable differences (Supplemental Figure S2).

**Table 1:** Impact of SnRK1 on Tre6P:sucrose ratios

Genotype	Time of day	Tre6P (nmol g^-1^ FW)	Sucrose (μmol g^-1^ FW)	Tre6P:Sucrose	*P*-value (versus control)	Reference
Col-0	ED	0.401 ± 0.08	2.083 ± 0.28	0.195 ± 0.04	NA	Figure 1 (*n *=* *8)
	EN	0.099 ± 0.02	0.843 ± 0.14	0.119 ± 0.03	NA	Figure 1 (*n *=* *10)
*SnRK1α1-OE*	ED	0.981 ± 0.31	2.584 ± 0.34	0.376 ± 0.10	6.00E−04	Figure 1 (*n *=* *8)
	EN	0.159 ± 0.05	0.939 ± 0.16	0.170 ± 0.04	1.15E−02	Figure 1 (*n *=* *10)
*sesquiα2*	ED	0.114 ± 0.01	1.678 ± 0.20	0.069 ± 0.01	2.00E−04	Figure 1 (*n *=* *8)
	EN	0.037 ± 0.02	0.749 ± 0.09	0.047 ± 0.03	3.00E−04	Figure 1 (*n *=* *10)

Genotype	Time of day	Tre6P (nmol g^-1^ FW)	Sucrose (μmol g^-1^ FW)	Tre6P:Sucrose	*P*-value (versus control)	Reference

Col-0	ED	0.417 ± 0.06	1.802 ± 0.29	0.232 ± 0.02	NA	Yadav et al. (*n *=* *5)
	EN	0.097 ± 0.01	0.729 ± 0.11	0.134 ± 0.00	NA	Yadav et al. (*n *=* *5)
*TPS-OE*	ED	2.388 ± 0.27	1.222 ± 0.11	1.955 ± 0.16	1.60E−02	Yadav et al. (*n *=* *4)
	EN	1.025 ± 0.34	0.362 ± 0.10	2.831 ± 0.53	7.90E−03	Yadav et al. (*n *=* *5)
*TPP-OE*	ED	0.399 ± 0.04	3.423 ± 0.52	0.118 ± 0.02	7.90E−03	Yadav et al. (*n *=* *5)
	EN	0.088 ± 0.02	0.995 ± 0.13	0.088 ± 0.01	7.90E−03	Yadav et al. (*n *=* *5)

Yadav et al., 2014; 12:12 conditions.

Ratios are means ± sd and were calculated using only ED (12 h and 36 h after first dawn), and EN (24 h and 48 h after first dawn) time points. *P*-values refer to statistically significant differences (Student’s *t* test) between Col-0 and each of the indicated SnRK1 mutants. These data are graphically represented in Figure 1C, and use the Tre6P and sucrose values of Figure 1, A and B. The values previously obtained for plants with altered Tre6P accumulation (*TPS-OE and TPP-OE*, 12:12 photoperiod, EN and ED) by Yadav et al. (2014) are also shown for comparison. FW, Fresh weight. Sucrose levels correspond to hexose equivalents.

The proposal that Tre6P is a signal of the sucrose status was originally prompted by the tight correlation observed between the levels of Tre6P and sucrose (Lunn et al., 2006). We therefore wondered whether manipulation of SnRK1 had any effect on the relationship between these two sugars and generated the corresponding sucrose–Tre6P regression plots to assess this. A strong positive correlation between Tre6P and sucrose was observed in all lines, with *R* values of 0.95, 0.94, and 0.86 in Col-0, *SnRK1α1-OE*, and *sesquiα2*, respectively (Figure 1C and Table 2). However, compared to Col-0, the slope of the regression was markedly lower in *SnRK1α1-OE* and higher in the *sesquiα2* mutant.

**Table 2:** Impact of SnRK1 on the Tre6P–sucrose relationship

Genotype	*R*	*P*-value	Slope
Col-0	0.95	2.60E−09	3.80
*SnRK1α1-OE*	0.94	5.00E−09	1.80
*sesquiα2*	0.86	3.90E−06	9.70

Genotype	*R*	*P*-value	Slope

Col-0 (Yadav et al.)	0.99	1.90E−07	3.40
*TPS-OE* (Yadav et al.)	0.97	1.90E−05	0.58
*TPP-OE* (Yadav et al.)	0.96	7.90E−06	7.70

The Tre6P content of Col-0, *SnRK1α1-OE*, and *sesquiα2* rosettes at ED (12 h and 36 h after first dawn), and EN (24 h and 48 h after first dawn) was plotted against the corresponding values of sucrose. *R*, Pearson correlation coefficient. Slope, slopes of the corresponding Tre6P–sucrose regression curves (Figure 1C). Data previously obtained for the *TPS-OE and TPP-OE* lines by Yadav et al. (2014) are also shown for comparison.

A previous study (Yadav et al., 2014) compared the diel levels of Tre6P and sucrose in three different photoperiods in plants constitutively expressing a bacterial TPS or TPP (*Escherichia coli* OtsA or OtsB; *TPS-OE and TPP-OE*, respectively). We retrieved the data from the 12:12 photoperiod of this study and re-calculated the average values at ED and EN (Table 1). The Tre6P:sucrose ratios of *TPS-OE* were higher than those of Col-0, qualitatively resembling *SnRK1α1-OE* plants. However, the differences in *TPS-OE* plants were much more pronounced than in *SnRK1α1-OE* (8.4- and 21.2-fold higher than in Col-0 at ED and EN, respectively), and were due to lower sucrose as well as increased Tre6P. In *TPP-OE*, Tre6P:sucrose ratios were, like the *sesquiα2* mutant, lower than in Col-0 plants (2- and 1.5-fold lower than in Col-0 at ED and EN, respectively), and were exclusively due to high sucrose.

We next asked what could be the cause for the changed Tre6P levels in the SnRK1 mutants. The magnitude of the SnRK1-dependent change in Tre6P levels was smallest when sucrose content reached its minimum at EN (1.6-fold higher in *SnRK1α1-OE* and 2.7-fold lower in *sesquiα2* versus Col-0), and largest when sucrose peaked at ED (2.4-fold higher in *SnRK1α1-OE* and 3.5-fold lower in *sesquiα2* versus Col-0). This is clearly seen in the corresponding sucrose:Tre6P regression plots (Supplemental Figure S3A), and suggests that SnRK1 affects the sensitivity of Tre6P synthesis and/or degradation to sucrose, leading to sucrose hypersensitivity of Tre6P in *SnRK1α1-OE* and sucrose hyposensitivity of Tre6P in the *sesquiα2* mutant.

**Figure 2:**
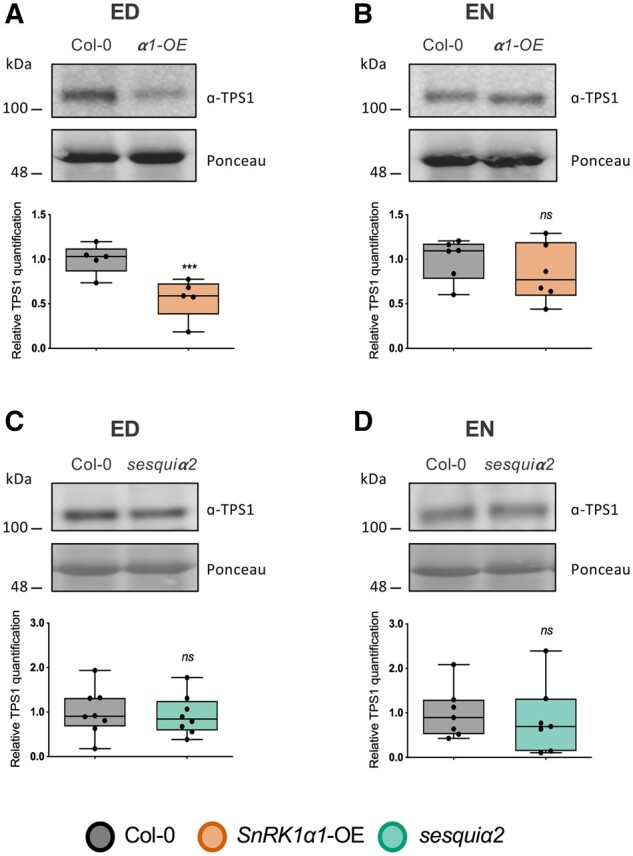
Impact of SnRK1 on TPS1 protein accumulation. Levels of TPS1 protein were quantified by immunoblot analyses in Col-0 control plants and *SnRK1α1-OE* (A, B) and *sesquiα2* (C, D) mutants at ED (A, C) and EN (B, D). Upper parts, representative immunoblots for the TPS1 protein. Lower parts, boxplots representing a minimum of four biological replicates (each consisting of a pool of three randomly harvested rosettes). Lower and upper box boundaries represent the first and third quantiles, respectively, horizontal lines mark the median and whiskers mark the highest and lowest values. Dots represent individual datapoints. *P*-values denote statistically significant differences between SnRK1 mutants and Col-0 at the indicated times of the day (paired *t* test); NS, nonsignificant; ***P <* *0.001.

To look further into the underlying mechanisms, we quantified the levels of TPS1, the predominant Tre6P-synthesizing enzyme in Arabidopsis (Delorge et al., 2015; Fichtner et al., 2020). Surprisingly, TPS1 levels were lower at ED in *SnRK1α1-OE* (Figure 2A), but no differences were observed at EN (Figure 2B). *TPS1* mRNA levels were unchanged (Supplemental Figure S3B). Given the high Tre6P accumulation of *SnRK1α1-OE*, decreased TPS1 levels could be due to product-mediated negative feedback, *via* translational regulation or stability of the TPS1 protein. For *sesquiα2*, no differences were observed compared to Col-0 (Figure 2, C and D).

Collectively, these results show that Tre6P and sucrose levels are affected in the SnRK1 mutants. Furthermore, the responsiveness of Tre6P to changes in sucrose is also altered in these plants, suggesting that SnRK1 links sucrose to Tre6P synthesis and/or degradation through mechanisms other than TPS1 accumulation.

### Impact on diel starch turnover

In all genotypes, starch showed qualitatively similar diel changes, being accumulated and mobilized in a near-linear manner during the day and night, respectively, and almost fully exhausted at EN (Supplemental Figure S4A). However, starch levels were moderately but significantly higher in *SnRK1α1-OE* and lower in *sesquiα2*, particularly toward the ED (Supplemental Figure S4A). Accordingly, the rate of starch accumulation was significantly lower in *sesquiα2* compared to Col-0 (Supplemental Figure S4B). The rates of degradation mirrored those of starch accumulation in all the genotypes, consistent with starch mobilization being determined by the amount of starch reserves and the length of the night (Graf et al., 2010; Scialdone et al., 2013). Compared to Col-0, sucrose levels and starch content increased in *SnRK1α1-OE* and decreased in *sesquiα2*. In both cases, the changes of sucrose were larger than those of starch. Hence, the starch:sucrose ratio was lower in *SnRK1α1-OE* and higher in *sesquiα2* (Supplemental Figure S4C), with differences being most significant at ED, when sucrose levels are most divergent in these mutants.

### Impact on sugar phosphates and organic acids

**Figure 3:**
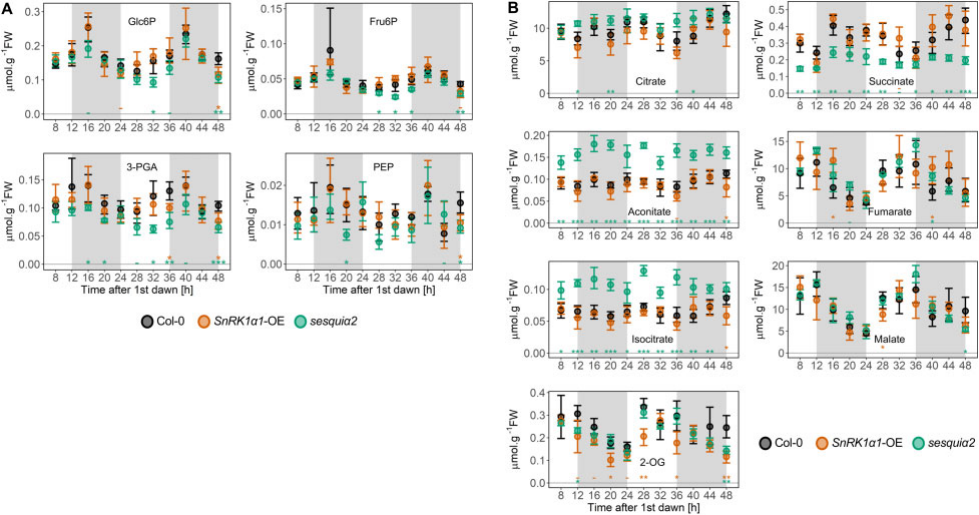
Impact of SnRK1 on the levels of glycolytic and TCA cycle intermediates. Levels of glycolytic (A) and TCA cycle intermediates (B) were quantified from 20-d-old Col-0, *SnRK1α1-OE*, and *sesquiα2* plants grown under a 12:12 photoperiod and harvested every 4 h. The night period is marked in gray. Graphs show average values for the same samples as in Figure 1 (*n *=* *4–5 biological replicates, each composed of a pool of four to five randomly sampled whole rosettes at each time point), with error bars representing the 95% confidence interval. Asterisks denote statistically significant differences tested at each ZT for both genotypes separately (one-way ANOVA with Tukey’s HSD post hoc test); ^-^*P* < 0.1 cases in which the Tukey’s HSD test resulted in nonsignificant differences; *P <* *0.05; **P <* *0.01; ***P <* *0.001. Glc6P, glucose 6-phosphate; Fru6P, fructose 6-phosphate; 3-PGA, 3-phosphoglycerate; PEP, phosphoenolpyruvate; 2-OG, 2-oxoglutarate.

The levels of sugar phosphates and glycolytic intermediates tended to be lower in *sesquiα2* than in Col-0, in particular in the case of fructose 6-phosphate (Fru6P) and 3-phosphoglycerate (3-PGA), for which differences were significant at four and five time points, respectively (Figure 3A;Supplemental Table S1, D and E). For Glc6P and phosphoenolpyruvate, differences reached significance only at two time points. Several tricarboxylic acid cycle (TCA) intermediates, citrate, aconitate, and isocitrate accumulated to significantly higher levels in *sesquiα2* at four, eleven, and ten time points, respectively. Remarkably, an opposite pattern was observed for succinate, which was significantly decreased in *sesquiα2* at ten time points. For 2-OG, a subtle yet significant reduction was also visible at two time points. Malate and fumarate showed inconsistent and mostly nonsignificant alterations.

In *SnRK1α1-OE*, the levels of sugar phosphates, glycolytic, and TCA cycle intermediates were mostly similar to Col-0. The only exception was 2-OG, which accumulated to significantly lower levels in *SnRK1α1-OE* at four time points (Figure 3, A and B; Supplemental Table S1, B and C).

### Impact on the transcriptome

**Figure 4:**
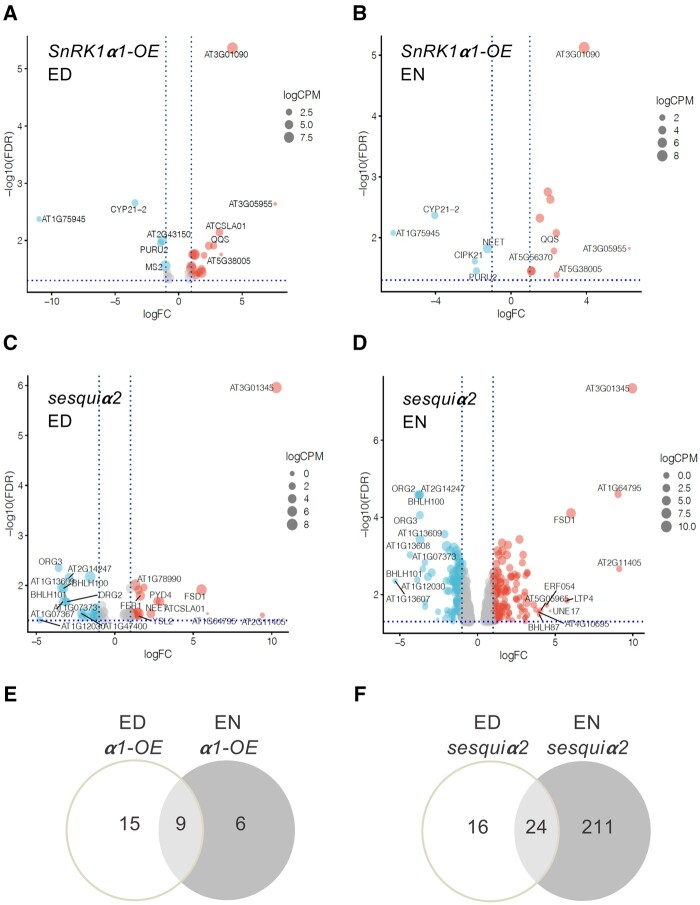
Impact of SnRK1 on the transcriptome. Volcano plot representation of RNA-seq analyses of the *SnRK1α1-OE* (A, B) and *sesquiα2* (C, D) mutants in comparison to Col-0 plants at ED (A, C) and EN (B, D). Up- and downregulated genes are reported as red and light blue dots, respectively. Gray dots represent genes whose differential expression is considered not significant (absolute log_2_ FC ≤ 1, vertical dotted blue lines, and FDR ≥ 0.05, horizontal dotted blue line). Comparison of the DEG lists for the *SnRK1α1-OE* (E) and *sesquiα2* (F) mutants reveals genes that are similarly affected at the ED and EN time points.

To investigate the relevance of SnRK1 for gene expression during the regular diel cycle, we extracted RNA from the samples collected for metabolite quantification at ED and EN (three replicates per genotype and condition; Supplemental Figure S1B) and performed RNA sequencing (RNA-seq) analyses. Volcano plots show differentially expressed genes (DEGs) for each pairwise comparison (*SnRK1α1-OE* versus Col-0 and *sesquiα2* versus Col-0) and time point (FDR < 0.05), revealing the greatest differences, by far, for *sesquiα2* at EN (Figure 4). For all subsequent comparisons we considered as DEGs those with an absolute log fold-change (FC) equal to or greater than 1 (ABSlogFC ≥ 1; two-fold change).

The *SnRK1α1-OE* mutant showed mild defects in transcript abundance, with only 24 (19 up- and 5 downregulated) and 15 (10 up- and 5 downregulated) DEGs at ED and EN, respectively (Figure 4, A and B; Supplemental Table S2, A–D). These genes showed very poor overlap with those induced by transient SnRK1α1 overexpression in protoplasts (Baena-González et al., 2007), with only At3g62550 (an adenine nucleotide alpha hydrolase-like protein), being induced in both conditions. This suggests that constitutive *SnRK1α1* overexpression in plants may trigger negative feedback mechanisms to attenuate SnRK1 activity.

We used MapMan (Thimm et al., 2004; Schwacke et al., 2019) categorization to assign these genes to specific MapMan BIN codes. At ED, from the genes upregulated in *SnRK1α1-OE* and with an assigned function, the largest differences were observed for *QUA-QUINE STARCH* (*QQS*), involved in the C:N balance (Li et al., 2015) and for *CELLULOSE SYNTHASE LIKE A1* (*CSLA1*). Additional upregulated genes included *IRON RESPONSIVE PROTEIN 3* (*IRP3*; Rodríguez-Celma et al., 2013). From the downregulated genes with assigned functions, the most prominent ones encoded *CYCLOPHILIN 21-2* (*CYP21-2*) and a 10-formyl tetrahydrofolate deformylase essential for photorespiration (*PURU2*; Collakova et al., 2008).

Eight of the genes that were differentially expressed at ED were also differentially expressed at EN (Supplemental Table S3A; Figure 4E), suggesting SnRK1α1 overexpression causes constitutive defects in their expression. Five of these were upregulated in *SnRK1α1-OE*, including *QQS*, and the other three were strongly repressed, including *PURU2 and CYP21-2*. Only six genes were differentially expressed in *SnRK1α1-OE* specifically at EN, including the downregulated gene *NEET*, which encodes a protein crucial for iron homeostasis (Nechushtai et al., 2012).

For the *sesquiα2* mutant, we identified a total of 40 DEGs at ED (18 up- and 22 downregulated) and 235 DEGs at EN (129 up- and 106 downregulated; Figure 4, C and D; Supplemental Tables S2, G and H). From the genes differentially expressed at ED, 60% also showed differential expression at EN (Supplemental Table S3B; Figure 4F), pointing to constitutive defects due to SnRK1 depletion. We analyzed these DEGs with AgriGOv2 (Tian et al., 2017) and found a significant overrepresentation of 16 functional categories, with the top gene ontology category being “iron ion homeostasis” (*P = *4.10E−10; Supplemental Table S4A). For the genes that were differentially expressed only in the EN or ED conditions, the top categories were “response to endogenous stimulus” (*P = *3.50E−12; Supplemental Table S4B), and “chemical homeostasis” (*P = *1.20E−06; Supplemental Table S4C), respectively.

From the 24 genes affected in *sesquiα2* both at ED and EN, 11 are part of a conserved set of genes responding to iron deficiency in leaves and roots (Rodríguez-Celma et al., 2013; Table 3). In all cases, genes downregulated in *sesquiα2* are induced by iron deficiency, while those upregulated in *sesquiα2* are repressed by iron deficiency. Although not comprised in the list of iron core response genes (Table 3), the depletion of SnRK1 led to defects both at EN and ED in other iron-responsive genes (Supplemental Table S2, E–H), including *IRONMAN 3* (*IMA3*), *IMA4*, *IMA6*, *BASIC HELIX-LOOP-HELIX 101* (*bHLH101*), and *YELLOW STRIPE-LIKE2* (*YSL2*). Other iron-responsive genes were affected specifically at EN (e.g. *NICOTIANAMINE SYNTHASE 1* [*NAS1*], *NAS3, ZINC TRANSPORTER 5 PRECURSOR* [*ZIP5*], and *IRON REGULATED TRANSPORTER 3* [*IRT3*]) or at ED (e.g. *NEET*, *HEMA1*, and *ZINC-INDUCED FACILITATOR1* [*ZIF1*]), altogether suggesting an iron excess response in the *sesquiα2* mutant.

**Table 3:** Expression of genes related to iron acquisition and metabolism in the *SnRK1* mutants

ID	Symbol	Description	Response to Fe deficiency^a^	*sesquiα2* ED	*sesquiα2* EN	*SnRK1α1-OE* ED
AT1G47400	*IRP1/IMA1*	IRON-RESPONSIVE PROTEIN 1 /IRONMAN 1	UP	−2.12	−3.20	–
AT1G47395	*IRP2/IMA2*	IRON-RESPONSIVE PROTEIN 2 /IRONMAN 2	UP	−2.07	−2.95	–
AT2G14247	*IRP3*	IRON-RESPONSIVE PROTEIN 3	UP	−2.91	−3.70	1.03
AT1G13609	*IRP4*	IRON-RESPONSIVE PROTEIN 4	UP	−3.38	−3.70	–
AT3G56360	*IRP5*	IRON-RESPONSIVE PROTEIN 5	UP	–	–	–
AT5G05250	*IRP6*	IRON-RESPONSIVE PROTEIN 6	UP	−1.22	−1.57	–
AT3G56970	*ORG2/bHLH38*	OBP3-RESPONSIVE GENE 2/BASIC HELIX-LOOP-HELIX PROTEIN 38	UP	−3.04	−3.83	–
AT3G56980	*ORG3/bHLH39*	OBP3-RESPONSIVE GENE 3/BASIC HELIX-LOOP-HELIX PROTEIN 39	UP	−3.58	−3.71	–
AT2G41240	*bHLH100*	BASIC HELIX-LOOP-HELIX PROTEIN 100	UP	−3.25	−3.76	–
AT1G23020	*FRO3*	FERRIC REDUCTION OXIDASE 3	UP	−1.92	−1.53	–
AT4G16370	*OPT3*	OLIGOPEPTIDE TRANSPORTER	UP	–	–	–
AT5G53450	*ORG1/PAP14*	OBP3-RESPONSIVE GENE 1	UP	−1.57	−1.11	–
AT1G56430	*NAS4*	NICOTIANAMINE SYNTHASE 4	UP	−1.34	–	–
AT4G08390	*SAPX*	STROMAL ASCORBATE PEROXIDASE	DOWN	–	–	–
AT4G25100	*FSD1*	FE SUPEROXIDE DISMUTASE 1	DOWN	5.53	6.02	–
AT5G01600	*FER1*	FERRETIN 1	DOWN	1.63	–	–
AT1G48300	*DGAT3*	DIACYLGLYCEROL ACYLTRANSFERASE 3	UP	–	–	–

a Rodríguez-Celma et al. (2013).

Expression levels of 17 core genes responsive to iron in both leaves and roots (Rodríguez-Celma et al., 2013; Table 1) in the indicated genotypes at ED and EN. The response of these genes to iron deficiency is indicated for comparison (UP, upregulated; DOWN, downregulated compared to control conditions). None of these genes were retrieved as DEGs in the *SnRK1α1-OE* at EN. Numbers indicate log2 ratios of *sesquiα2* compared to the Col-0 control.

At ED only a few genes unrelated to iron metabolism were affected in *sesquiα2* (Supplemental Table S2, E and F), including *CSLA1*, which was surprisingly upregulated as in *SnRK1α1-OE.*

At EN, a clear impact was also observed in sulfur-responsive genes (Supplemental Table S2, G and H). From the 20 genes reported to be most responsive to sulfur deprivation in leaves (Forieri et al., 2017), 8 were affected in the *sesquiα2* rosettes (Table 4). These genes are induced by sulfur deprivation and, in all but one of the cases (At1g12030), they were upregulated in the *sesquiα2* mutant. In addition, *REVEILLE 2* (*RVE2*), repressed by sulfur deficiency in roots (Forieri et al., 2017), was downregulated in *sesquiα2*, altogether suggesting a sulfur starvation response when SnRK1 is depleted.

**Table 4:** Expression of genes related to sulfur acquisition and metabolism in the *sesquiα2* mutant

ID	Symbol	Description	Response to S deficiency^a^	*sesquiα2* ED	*sesquiα2* EN
AT5G48850	*ATSDI1*	SULFUR DEFICIENCY-INDUCED 1	UP (leaves)	–	1.95
AT3G49580	*LSU1*	RESPONSE TO LOW SULFUR 1	UP (leaves)	–	2.14
AT3G49570	*LSU3*	RESPONSE TO LOW SULFUR 3	UP (leaves)	–	2.17
AT5G26220	*GGCT2;1*	GAMMA-GLUTAMYL CYCLOTRANSFERASE 2;1	UP (leaves)	–	2.21
AT3G08860	*PYD4*	PYRIMIDINE 4	UP (leaves)	2.69	3.21
AT4G35640	*SERAT3;2*	SERINE ACETYLTRANSFERASE 3;2	UP (leaves)	–	1.04
AT4G31330		Putative transmembrne protein (DUF599)	UP (leaves)	–	1.21
AT1G12030		Putative phosphoenolpyruvate carboxylase (DUF506)	UP (leaves)	−4.78	−3.88
AT5G37260	*RVE2*	REVEILLE 2	DOWN (roots)	–	−1.25

a Forieri et al. (2017).

Expression levels of genes reported to be most responsive to sulfur deprivation (Forieri et al., 2017; Supplemental Table S4) in the *sesquiα2* mutant at ED and EN. Numbers indicate log2 ratios of *sesquiα2* compared to the Col-0 control.

Despite the metabolic differences caused by SnRK1 depletion in *sesquiα2* (Figures 1 and 3), genes related to primary metabolism remained mostly unaffected, with the notable exception of *TPPD*, *TPPG*, *TPPH*, and *TPPJ*, which were all upregulated at EN. Given the potential relevance of this finding for the observed Tre6P phenotype (Figure 1B), we sought to confirm these results for selected *TPP* genes by reverse transcription quantitative polymerase chain reaction (RT-qPCR), including also the ED and the *SnRK1α1-OE* samples (Supplemental Figure S5A). These analyses corroborated the RNAseq results, revealing a significant upregulation of *TPPG*, *TPPH*, and *TPPJ* in *sesquiα2* specifically at EN. In the *SnRK1α1-OE* mutant, these *TPP* genes were in most cases mildly downregulated compared to Col-0 but the differences did not reach statistical significance.

**Figure 5:**
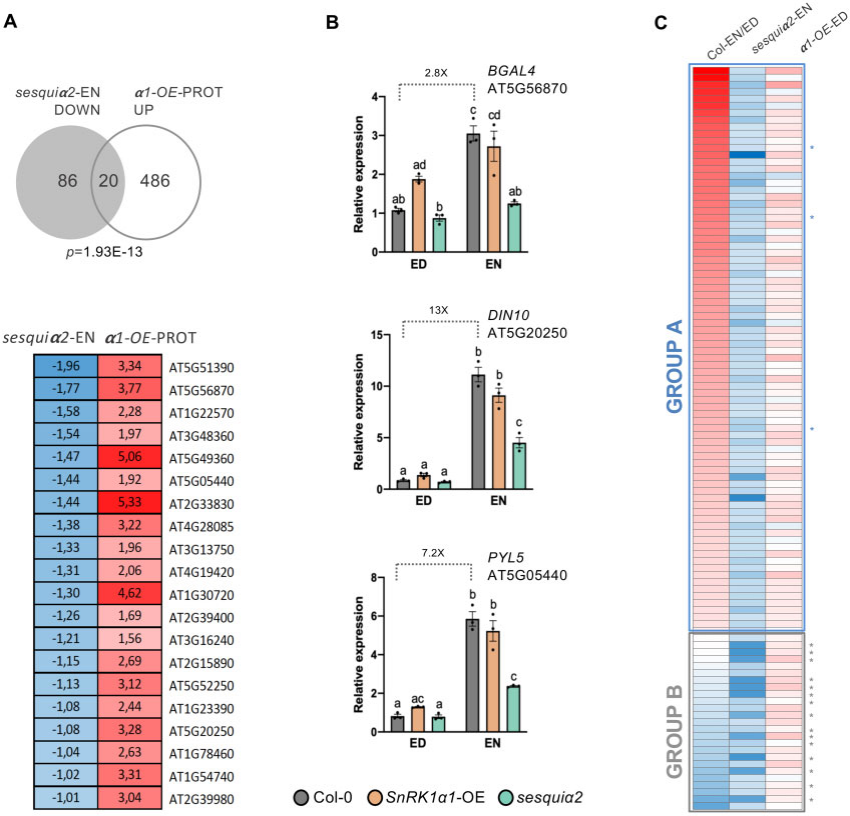
Basal expression of SnRK1-regulated genes at the ED and EN periods. A, Upper panel, overlap of genes downregulated in the *sesquiα2* mutant at EN (“*sesquiα2*-EN DOWN”) and those induced by transient SnRK1α1 overexpression in protoplasts (“α1-OE-PROT UP”; Baena-González et al., 2007). *P*-value denotes statistical significance (hypergeometric test). Lower panel, expression values (log_2_FC) of the overlapping genes in each of the indicated datasets. Red indicates upregulation and blue, downregulation. B, RT-qPCR analyses of genes (*BGAL4*, *DIN10*, and *PYL5*) from the overlapping set in (A) in rosettes of the indicated genotypes at ED and EN. Graphs correspond to the average of three independent experiments (error bars, sem (Standard Error of the Mean)). Letters indicate significantly different groups assessed using the ANOVA and Tukey’s HSD post test. C, Genes downregulated in the *sesquiα2* mutant at EN (“*sesquiα2*-EN DOWN”) are mildly upregulated in *SnRK1α1-OE* at ED. In Col-0, many of these genes are upregulated at EN versus ED (Group A, blue rectangle; asterisks correspond to *BGAL4*, *DIN10*, and *PYL5*). A smaller group of genes show either unchanged or lower expression in Col-0 at EN versus ED (Group B, gray rectangle). Asterisks correspond to the following genes, retrieved as DEGs in *sesquiα2* at ED (see Supplemental Table S3b): *IMA1/IRP1, IMA2/IRP2, IMA3, IMA4, IMA6, IRP3, IRP4, IRP6, PAP14/ORG1, BHLH38/ORG2, BHLH39/ORG3, BHLH100, BHLH101, FRO3*, AT1G12030.

We noticed a significant overlap between the genes downregulated in *sesquiα2* at EN and those previously reported to be induced by transient SnRK1α1 overexpression in protoplasts (Baena-González et al., 2007; Figure 5A;*P *=* *1.93E−13, hypergeometric test). To confirm this, we analyzed several of the overlapping genes (*BETA-GALACTOSIDASE 4* [*BGAL4*], *DARK-INDUCED 10* [*DIN10*], and *PYRABACTIN RESISTANCE 1-LIKE 5* [*PYL5*]) by RT-qPCR, including also the ED and the *SnRK1α1-OE* samples (Figure 5B). As controls, we included genes that were affected specifically in *SnRK1α1-OE* or *sesquiα2* in both time points (Supplemental Figure S5B). These analyses revealed lower expression of *BGAL4*, *DIN10*, and *PYL5* in the *sesquiα2* mutant at EN compared to Col-0, confirming the RNAseq results (Figure 5B). Interestingly, in Col-0, these genes had low expression at ED but were induced at EN, consistent with their activation by SnRK1α1 overexpression (Baena-González et al., 2007). Furthermore, at ED, these genes tended to have higher expression in *SnRK1α1-OE* than in Col-0, although differences were not significant. Other genes originally identified as markers of SnRK1 signaling (Baena-González et al., 2007) showed a similar pattern of expression, but the differences were again small, explaining why they were not retrieved as DEGs in the RNAseq analysis (Supplemental Figure S5C).

To assess more broadly the behavior of the DEGs downregulated in *sesquiα2* at EN, we compared their expression levels in *SnRK1α1-OE* versus Col-0 at ED and in Col-0 at EN versus ED (Figure 5C). In general, all of these genes showed a mild upregulation in *SnRK1α1-OE* at ED. However, they fell into two distinct groups with regard to their expression in Col-0. Group A included genes like *BGAL4*, *DIN10*, and *PYL5* (blue asterisks in Figure 5C), with higher expression at EN than at ED in Col-0 and with decreased levels at EN in *sesquiα2* compared to Col-0. This suggests that SnRK1 is normally activated towards the EN, triggering amongst others the induction of these genes. Group B contained many of the genes previously identified as iron-responsive (Table 3; gray asterisks in Figure 5C). Their expression was higher at ED than at EN in Col-0, but they appeared to be constitutively downregulated in *sesquiα2* (EN and ED). This suggests that the effect of SnRK1 on these genes is more indirect than on Group A genes, and is also independent of time-of-day.

### Diel fluctuations in Tre6P levels can modulate SnRK1 activity

**Figure 6:**
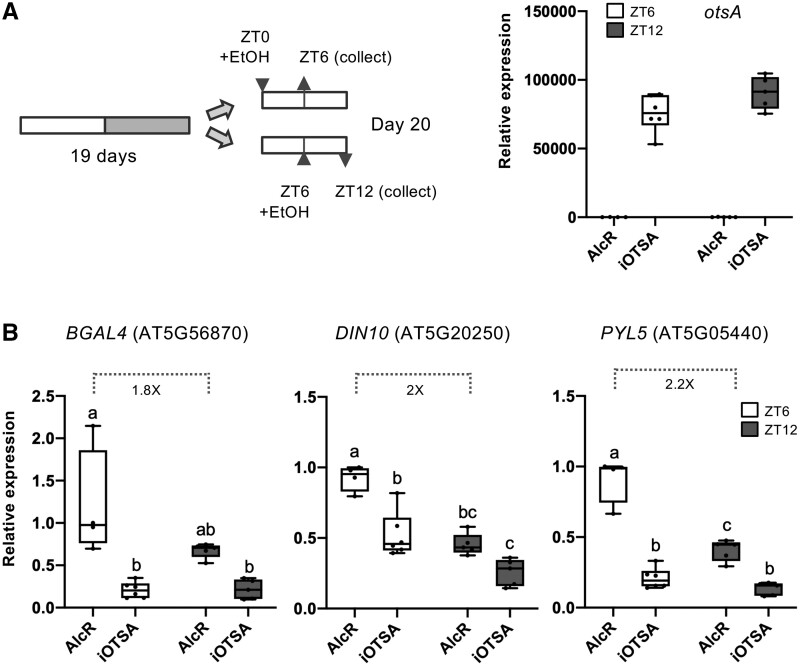
Impact of Tre6P on the basal expression of SnRK1-regulated genes. A, Induction of otsA expression in an ethanol-inducible line (iOtsA) and the corresponding empty vector control (AlcR). Plants were grown on soil, under a 12:12 photoperiod for 19 d. Ethanol (2% v/v) was sprayed over the plants at ZT0 or at ZT6 and whole rosettes were harvested after 6 h (ZT6 and ZT12 indicate the time of harvest) for RT-qPCR analyses of otsA (A, right) and of the same set of SnRK1 marker genes as in Figure 5B (B). Values denote expression relative to the ZT6 time point of the AlcR line. Boxplots represent four to five biological replicates (each consisting of a pool of three randomly harvested rosettes). Lower and upper box boundaries represent the first and third quantiles, respectively, horizontal lines mark the median and whiskers mark the highest and lowest values. Dots represent individual datapoints. Different letters indicate statistically significant differences (*P *<* *0.05, one-way ANOVA with Tukey’s HSD test).

The expression of Group A genes was highest when Tre6P levels are low at EN and lowest when Tre6P levels are high at ED (Figures 5, B and C, 1B), prompting the hypothesis that Tre6P build-up during the day inhibits SnRK1 activity at ED. If true, we reasoned that inducing Tre6P accumulation should repress these genes during the day. To test this, we used an ethanol inducible OtsA (iOtsA) line in which OtsA expression drives Tre6P production, and an AlcR control line, harboring the corresponding empty vector (Martins et al., 2013). AlcR and iOtsA plants were sprayed with ethanol at ZT0 or ZT6 and harvested 6 h later, at ZT6 (middle of the day, MD) and ZT12 (ED). The expression of *otsA* in all iOtsA samples was confirmed by RT-qPCR (Figure 6A). Group A genes had higher expression in the AlcR control line at MD than at ED (Figure 6B), showing that the high expression uncovered in the RNAseq analyses for Col-0 at EN extends into the first half of the day. However, the differences between EN and ED (Figure 5B) were clearly larger than those between MD and ED (Figure 6B), supporting the idea that these genes are increasingly repressed as the day progresses. In contrast, OtsA induction in the iOtsA line resulted in a marked repression of these genes (Figure 6B). The repression tended to be larger at MD, when the expression of these genes in the AlcR control line was higher, but was also visible at ED. For genes whose expression was already very low towards the ED, Tre6P accumulation did not have any further repressive effect (Supplemental Figure S5D).

## Discussion

Our results show that SnRK1 plays important functions in the regulation of sucrose homeostasis and the transcriptome under standard laboratory growth conditions that are favorable for Arabidopsis. They further suggest that diel oscillations in Tre6P levels contribute to the regulation of basal SnRK1 activities during the regular day–night cycle, consistent with a recent study linking diel oscillations in sucrose and SnRK1 activity with rhythms of nitrogen accumulation in maize (*Zea mays*) seeds (Li et al., 2020).

### SnRK1 modulates the Tre6P–sucrose relationship

According to the sucrose–Tre6P nexus model, Tre6P is essential for sucrose homeostasis, acting both as a signal of sucrose availability, and as a feedback regulator of sucrose production and consumption (Yadav et al., 2014; Figueroa and Lunn, 2016). The ratio of Tre6P:sucrose is important for maintaining sucrose levels within an adequate range, with high Tre6P:sucrose ratios in actively growing tissues being thought to promote sucrose utilization and import (Lunn et al., 2014; Yadav et al., 2014; Fichtner and Lunn, 2021). In our study with Arabidopsis rosettes, SnRK1α1 overexpression and depletion resulted in increased and decreased Tre6P:sucrose ratios, respectively (Table 1). While being initially surprising in view of the known growth-inhibitory functions of SnRK1, these results are not unexpected because SnRK1 is also required for growth (Margalha et al., 2019; Baena-González and Lunn, 2020).

Although Tre6P and sucrose were still highly correlated in the SnRK1 mutants (Figure 1C), their relationship was clearly altered, as evidenced by the slopes of the Tre6P:sucrose regression lines (Table 2). SnRK1α1 overexpression may cause “Tre6P hyposensitivity” partly by blocking the effect of Tre6P on sucrose metabolism, as suggested by the rather weak effect of high Tre6P on organic acid levels in *SnRK1α1-OE* (Table 1 and Figure 3B). Conversely, SnRK1α depletion may cause “Tre6P hypersensitivity”, allowing changes in sucrose metabolism in response to Tre6P levels that are lower than in control plants. Accordingly, we observed an enhanced accumulation of citrate, aconitate, and isocitrate in *sesquiα2* (Figure 3B), suggesting that the flux of photoassimilates into the TCA cycle increases or that consumption of these tricarboxylic acids decreases when SnRK1 is depleted. This is the opposite of what would be expected for a plant with low Tre6P, given the increased organic acid content of iOtsA lines with elevated Tre6P (Figueroa et al., 2016), and may indicate that the reported effects of Tre6P on sucrose utilization rely at least partly on inhibition of SnRK1. This may involve stimulation of anaplerotic reactions through PEPC, as shown for Tre6P (Figueroa et al., 2016), but could also involve alternative or additional mechanisms. Interestingly, the higher levels of citrate, aconitate, and isocitrate of the *sesquiα2* mutant were accompanied by lower levels of 2-OG and, in particular succinate. One possible explanation for such a differential impact on TCA cycle intermediates is that there is an increased diversion of 2-OG toward nitrogen assimilation and/or photorespiration when SnRK1 is depleted. This is consistent with the known inhibitory effect of SnRK1 on NR activity (Sugden et al., 1999; Jossier et al., 2009; Li et al., 2009). The observed downregulation of *PURU2*, essential for photorespiration (Collakova et al., 2008), in the *SnRK1α1-OE* mutant, may on the other hand support a link between SnRK1 and photorespiration.

Our results with the *sesquiα2* mutant are in agreement with the low sucrose and high organic acid content reported for an inducible *snrk1* knockdown during an extended night treatment (Nukarinen et al., 2016). However, they are opposite to those reported for silenced SnRK1 in pea (*Pisum sativum*) embryos, potentially due to differences between source and sink tissues or to secondary effects derived from severe SnRK1 depletion and consequent embryo growth arrest (Radchuk et al., 2006, 2010). Opposite effects on sucrose and organic acids were also reported for a *snrk1β1* mutant (Wang et al., 2020). However, whether these effects are caused by the lack of SnRK1β1 itself or from compensatory effects from other β-subunits, remains to be determined.

Although SnRK1 had a strong impact on sucrose and especially on Tre6P, it had a much smaller effect on starch, suggesting that the latter is an indirect effect. The slight but significantly higher starch in *SnRK1α1-OE* and lower starch in *sesquiα2* (Supplemental Figure S4, A and B) is consistent with decreased and increased use of fixed carbon, respectively, and might reflect restricted starch mobilization by elevated Tre6P in *SnRK1α1-OE* and faster mobilization in *sesquiα2*, with lower Tre6P. The fact that we actually observed the opposite (slower starch mobilization in *sesquiα2*; Supplemental Figure S4B), may suggest that SnRK1 mediates the effects of Tre6P on starch mobilization. Alternatively, these effects on the rate of starch mobilization may be unrelated to Tre6P and simply mirror changes in the amount of starch accumulated during the day (Scialdone et al., 2013). The faster starch accumulation in *SnRK1α1-OE* and slower starch accumulation in *sesquiα2* might itself be an indirect response to the higher and lower sucrose levels. It is well-established that sucrose synthesis is subject to feedback regulation mediated by post-translational inhibition of sucrose–phosphate synthase, recycling of sucrose *via* reducing sugar to hexose phosphates, and an increase in the level of fructose 2,6-bisphosphate which inhibits cytosolic fructose-1,6.bisphophase (Stitt et al., 2010). This results in increased allocation to starch when sucrose is high.

### SnRK1 modulates the sucrose–Tre6P relationship

The main cause for the altered Tre6P:sucrose ratios of the SnRK1 mutants was a change in Tre6P levels (Figure 1, A and B and Table 1). Furthermore, Tre6P accumulation was not constitutively altered in the SnRK1 mutants but instead followed the dynamics of sucrose accumulation with a modified relationship. This observation points to SnRK1 being an important part of the regulatory network that connects Tre6P levels to the sucrose status. Thus, increased SnRK1 activity (as in *SnRK1α1-OE*) leads to sucrose hypersensitivity and a proportionally higher level of Tre6P than in Col-0 plants for the same amount of sucrose, and decreased SnRK1 activity (as in *sesquiα2*) leads to the opposite behavior. Such interpretation is in accordance with the sugar hypersensitivity of *SnRK1α1-OE* seedlings during early development, manifested as delayed germination and reduced growth under sugar concentrations that do not affect the wild-type (Baena-González et al., 2007; Rodrigues et al., 2013).

It is presently unclear how information on the sucrose status is conveyed *via* SnRK1 and other routes to regulate Tre6P levels, but could potentially involve altered Tre6P synthesis by TPS1, altered Tre6P dephosphorylation by TPP proteins or both. The modified Tre6P content of SnRK1 mutants could not be explained by changes in TPS1 protein abundance, which was unaltered or even decreased in the case of *SnRK1α1-OE* (Figure 2). Thus, if Tre6P accumulation is primarily determined by TPS1 activity, other mechanisms may be involved, such as synthesis of additional uncharacterized proteins or post-translational modification of TPS1 (Yadav et al., 2014; Fichtner et al., 2020). The lower TPS1 protein abundance in *SnRK1α1-OE* at ED (Figure 2A) suggests that the high Tre6P in this mutant may trigger negative feedback regulation on TPS1 *via* translational regulation or stability of the TPS1 protein. Such mechanisms might become apparent only when plants are exposed to high Tre6P levels over long periods, as in *SnRK1α1-OE*, because Tre6P accumulation in response to sucrose treatment did not alter TPS1 abundance within 3 h of supplying sucrose (Yadav et al., 2014). Although the low Tre6P levels of *sesquiα2* were accompanied by increased expression of four *TPP* genes at EN (Supplemental Table S2G and Supplemental Figure S5A), *SnRK1α1-OE* plants showed only mild nonsignificant downregulation of *TPP* expression (Supplemental Figure S5A). This suggests that transcriptional regulation of TPPs may not be the main mechanism by which SnRK1 affects Tre6P levels. Analyses of TPP protein abundance and activity in *SnRK1α1-OE and sesquiα2* mutants will therefore be needed in the future to assess if altered TPP activities contribute to the altered accumulation of Tre6P in these lines.

### SnRK1 impacts iron and sulfur metabolism genes

Very few of the DEGs in the SnRK1 mutants were related to metabolism, suggesting that the metabolic alterations observed in these plants involve post-transcriptional rather than transcriptional regulation. In contrast, SnRK1 had a profound impact on genes related to iron and sulfur acquisition and homeostasis, with iron metabolism genes being strongly downregulated in *sesquiα2* plants both at ED and EN and sulfur metabolism genes being upregulated in *sesquiα2* plants at EN.

Iron acquisition and metabolism are repressed by sulfur deprivation and *vice versa*, possibly as a way to coordinate the synthesis of Fe-S clusters (Forieri et al., 2013, 2017; Mendoza-Cózatl et al., 2019). Considering this, our interpretation is that in the *sesquiα2* mutant, iron acquisition may be aberrantly high, generating a demand for sulfur to match the iron levels and the consequent induction of sulfur starvation genes. Two observations support the hypothesis that the primary defect of *sesquiα2* might be in iron metabolism. First, the defects in iron responsive genes appear to be constitutive (visible at ED and EN) while those related to sulfur assimilation are specific to EN. Secondly, from the only four genes that were oppositely affected in *SnRK1α1-OE* and *sesquiα2*, two were related to iron metabolism (*IRP3 and NEET*).

### Tre6P affects SnRK1 activity in mature rosette leaves

SnRK1 is thought to be activated when energy (carbon) is low whilst Tre6P increases when energy in the form of sucrose is high. Accordingly, similar phenotypes could be expected from genetic manipulations that increase SnRK1 activity and those that decrease Tre6P levels while similar phenotypes could also be expected from treatments that decrease SnRK1 activity and those that increase Tre6P levels. In agreement with these expectations, *SnRK1α1-OE* plants show metabolic (e.g. high sucrose) and developmental phenotypes such as delayed flowering (Williams et al., 2014; Jeong et al., 2015) that are similar to those reported for *TPP-OE* (Avonce et al., 2004; Yadav et al., 2014). In turn, SnRK1 loss-of-function mutants show metabolic (low sucrose and higher levels of some organic acids) and developmental phenotypes such as early flowering (Tsai and Gazzarrini, 2012; Filipe et al., 2018), reported for *TPS-OE* and Tre6P accumulation (Avonce et al., 2004; Wahl et al., 2013; Figueroa et al., 2016).

However, these expected responses occur despite the contrasting pattern of Tre6P accumulation in *SnRK1α1-OE* versus *TPP-OE* (higher versus unchanged compared to the wild-type) and in *sesquiα2* versus *TPS-OE* (lower versus higher compared to the wild-type), suggesting that SnRK1 mediates at least some of the effects of Tre6P on these processes. This is further supported by (1) the negative correlation between Tre6P accumulation (Figure 1B) and the expression of a set of genes that are induced in a SnRK1-dependent manner at EN (Figure 5); (2) the fact that more genes were identified as differentially expressed between Col-0 and *SnRK1α1-OE* at ED than at EN, whereas the opposite was true for the *sesquiα2* mutant, for which the number of DEGs at EN far exceeded those at ED; (3) the fact that the expression of these genes during the day is substantially repressed by otsA induction (Figure 6). Altogether this suggests that under favorable conditions SnRK1 undergoes repression towards the ED and activation toward the EN and that these changes in SnRK1 activity are at least partly driven by diel changes in Tre6P accumulation. In plants grown under equinoctial conditions, the expression of SnRK1-dependent genes increases during the night (Bläsing et al., 2005; Frank et al., 2018), and increases strongly in an extended night, in the regular night of the starchless *PHOSPHOGLUCOMUTASE* mutant *pgm* (Usadel et al., 2008) or in the day and night after transfer to low irradiance (Moraes et al., 2019). This pattern of expression was attributed to progressive depletion of carbon reserves during the night. Moreover, several of these genes, including *PYL5 and DIN10*, show defective expression at EN in a knockout mutant of bZIP63 (Viana et al., 2021), a transcription factor targeted by SnRK1 (Mair et al., 2015), further supporting the view that the peak in the expression of these genes towards the EN requires SnRK1 signaling. Interestingly, the expression of a *LUCIFERASE* (*LUC*) reporter for the promoter activity of a SnRK1 marker gene (*DIN6::LUC*) declined rapidly during the first half of the night (Frank et al., 2018), suggesting that maximal accumulation of the endogenous transcript at EN may require mechanisms beyond mere transcriptional induction.

Our conclusion that SnRK1 mediates part of the Tre6P effects on metabolism and gene expression in whole Arabidopsis rosettes, appears to be in conflict with *in vitro* assays in which Tre6P inhibits SnRK1 activity only when extracted from actively growing tissues (Zhang et al., 2009; Nunes et al., 2013). This may be explained by the inability of *in vitro* kinase assays to detect moderate changes in SnRK1 activity (Zhang et al., 2009; Nunes et al., 2013) that can be amplified *in vivo*, leading to clear downstream effects on metabolite and transcript accumulation. Also, as compared to young seedlings, the proportion of SnRK1 that is susceptible to Tre6P inhibition may be much lower in mature leaves and hence appear negligible when both systems are compared. It is also possible that Tre6P affects not only SnRK1 activity but also other aspects of SnRK1 signaling, that are not addressed in *in vitro* activity assays, or that the Tre6P inhibition requires additional factors that are better captured in *in vitro* assays with extracts from sink tissues than from source leaves. Nevertheless, we cannot rule out the possibility that the observed effects are caused by another metabolite in a Tre6P-dependent manner rather than by Tre6P itself.

Although we have thus far mostly considered potentially direct effects of SnRK1 on metabolism and gene expression, it is plausible that some of these effects are indirect, caused by perturbations in the circadian clock. The overexpression of SnRK1α1 was shown to lengthen the circadian period and to delay the circadian phase (Shin et al., 2017; Frank et al., 2018). SnRK1 could alter the clock through the transcription of *RVE2*, the only clock-related component retrieved in our transcriptional analyses (Table 4; Zhang et al., 2007) and whose transcript abundance was shown to respond to low carbon conditions (Moraes et al., 2019). The effect of SnRK1 may also involve the action of the bZIP63 transcription factor (Frank et al., 2018; Viana et al., 2021), CRYPTOCHROME 1 (CRY1; Yuan et al., 2016) or TIME FOR COFFEE (TIC; Shin et al., 2017), a circadian regulator that affects numerous metabolic outputs (Sanchez-Villarreal et al., 2013) and has been implicated in iron homeostasis (Duc et al., 2009).

In conclusion, SnRK1 plays central functions in metabolic and transcriptional regulation under favorable conditions and this is modulated by diel fluctuations in Tre6P levels. SnRK1 is part of the system that translates changes in sucrose levels into changes in Tre6P. SnRK1 also mediates or modulates the effects of Tre6P on the TCA cycle. Overall, the effects of SnRK1 depletion on metabolism and transcript abundance were considerably stronger than those of SnRK1 overexpression, suggesting constitutive SnRK1 activation is largely dampened by compensatory negative feedback mechanisms or that additional components (e.g. regulatory subunits) are required for inducing such responses.

## Materials and methods

A list of all primers, antibodies and plant lines used in this study is provided in Supplemental Table S5.

### Plant material and growth conditions

All *A. thaliana* plants used here are in the Columbia (Col-0) background. The *sesquiα2* mutant bears the *snrk1α1-3* mutation (GABI_579E09) in homozygosity and the *snrk1α2-2* mutation (WiscDsLox384F5) in heterozygosity and was previously described (Belda-Palazón et al., 2020). The *SnRK1α1-OE*, AlcR and iOtsA lines were described elsewhere (Jossier et al., 2009; Martins et al., 2013).

Seeds were sown in excess directly on a 1:1 mix of soil (Stender; www.stender.de) and vermiculite (soaked with tap water, supplemented with boron [1.8 mg L^−1^] and the fungicide Previcur [1.5 mL L^−1^; Bayer; www.bayer.de]) in round plastic pots (10-cm diameter). Pots were thereafter placed in a cold room (4° C) for 2–3 d before being transferred to growth cabinets (Percival E-36 L, CLF Plant Climatics GmbH, Wertingen, Germany) with the following settings: 12:12 light:dark photoperiod, 22:18° C temperature regime, 60% RH, and 160 µmol m^−2^ s^−1^ irradiance provided by white fluorescent tubes giving a light spectrum as described (Annunziata et al., 2017). The pots were covered with transparent plastic lids during the first 5 d and 2 d later the plants were thinned out to leave three to five plants per pot. All pots were well separated to prevent shading and their positions were randomized every 3 d to avoid positional effects. Plants were watered twice a week to ensure soil humidity. To this end, water was added to the trays and excess water (not absorbed by the pots) was removed from the trays after 1 h. To avoid pests, plants were treated weekly with *Caenorhabditis elegans* nematodes. Plant harvesting and treatments were started always in the light period of the 20th d of growth. The *sesquiα2* mutant was always pre-selected on 0.5 × MS medium supplemented with BASTA (10 µg mL^−1^) for 6 d and seedlings were thereafter transferred to soil. For ethanol-treated plants (AlcR and iOtsA), a solution of 2% (v/v) ethanol was gently sprayed over the rosettes, 6 h prior to harvest. Whole rosettes were harvested, flash frozen in liquid nitrogen and stored at −80°C until further analyses.

### Metabolite extraction and determination

For metabolite analyses, Col-0, *SnRK1α1-OE*, and *sesquiα2* plants were harvested at 4-h intervals for a period of 40 h starting at ZT8.

For enzymatic methods, approximately 20 mg of finely ground plant material was extracted twice in boiling 80% (v/v) ethanol and once in 50% (v/v) ethanol, both buffered with 10-mM HEPES-NaOH (pH 7.0). The resulting supernatants were used to measure soluble sugars (glucose, fructose, and sucrose) enzymatically, as described (Stitt et al., 1989). NO_3_^−^ and NO_2_^−^ amounts were also enzymatically determined from the ethanolic extracts (Cross et al., 2006). Both starch and total protein contents were determined from the insoluble material left after ethanol extraction, as described (Hendriks et al., 2003).

For LC–MS/MS-based quantifications, ∼20 mg of finely ground plant material was quenched in a 3:7 (v/v) chloroform:methanol mixture at liquid nitrogen temperature, and allowed to warm up to −20°C for 2 h. The chloroform phase was then extracted twice with ice-cold ultra-pure water. The two water phases were pooled together and evaporated to dryness in a centrifugal vacuum drier at 20°C. Pellets were resuspended in 350 µL of ultra-pure water, filtered through a Multiscreen Ultracel-10 (Milipore) membrane to remove high molecular weight components, and diluted 1:10 before analysis by high-performance anion-exchange chromatography coupled to tandem mass spectrometry, as described in (Lunn et al., 2006) with modifications (Figueroa et al., 2016).

### Protein extraction and TPS1 immunoblotting

For the extraction of total protein, whole rosettes (three per replicate) were ground in liquid N_2_. Finely ground tissue (∼30 mg per sample) was extracted in 1.5 volumes of buffer (50-mM NaCl, 1% [v/v] Igepal CA-630, 0.5% [w/v] sodium deoxycholate, 0.1% [w/v] sodium dodecyl sulphate, 50-mM Tris–HCl [pH 8.0], 1-mM EDTA [pH 8.0], supplemented with 50-µM MG132, 50-mM *N*-ethyl maleimide, complete EDTA-free Protease Inhibitor Cocktail [Roche; 1 tablet/20 mL], and 1:500 Phosphatase Inhibitor Cocktails 2 and 3 [Sigma-Aldrich]). Homogenates were cleared by centrifugation at 21,130*g* for 15 min at 4°C, and supernatants were used for protein quantification (Pierce 660 nm protein assay). Single use aliquots of 40-µg total protein were prepared and denatured in Laemmli buffer (Laemmli, 1970) before storage at −20°C. Total protein samples were separated by sodium dodecyl sulphate–polyacrylamide gel electrophoresis in 7% (w/v) acrylamide gels, transferred to polyvinylidene difluoride membranes (wet transfer at 4°C, 110 V, 60 min) and probed using a 1:3,000 dilution of an anti N-terminal TPS1 antibody (Yadav et al., 2014). Chemiluminescent detection was performed using a 1:10,000 dilution of Peroxidase AffiniPure goat anti-rabbit IgG (H + L; Jackson ImmunoResearch), and SuperSignal West Femto Maximum Sensitivity Substrate (Thermo Scientific).

### RNA extraction and RT-qPCR

Total RNA was extracted from ∼50 mg of finely ground tissue, with the Plant Nucleospin RNA extraction kit (Macherey-Nagel), following the manufacturer’s instructions. DNAse-treated RNA (0.5 µg) was used for cDNA synthesis, using SuperScript III Reverse Transcriptase (Life Technologies), as previously described (Rodrigues et al., 2013). qPCR was performed in a QuantStudio 7 platform (Applied Biosystems), with the i*Taq* Universal SYBR Green Supermix (Bio-Rad), and the 2^−^^ΔΔCt^ method for relative quantification (Livak and Schmittgen, 2001). The expression values of SnRK1-induced genes were normalized using the geometric mean of *UBIQUITIN 10* (*UBQ10*), *UBIQUITIN-CONJUGATING ENZYME 21* (*UBC21*), and *EUKARYOTIC INITIATION FACTOR 4A* (*EIF4A*; Vandesompele et al., 2002).

### RNA-seq analysis

For RNA-seq analyses, Col-0, *SnRK1α1-OE*, and *sesquiα2* rosettes were harvested at the ED and EN. Three biological replicates (each composed of a pool of four to five randomly sampled whole rosettes) were generated from each genotype and time point. Total RNA was extracted from ∼50 mg of finely ground tissue, with the Qiagen RNeasy Plant Mini Kit following the manufacturer’s instructions. RNA integrity was confirmed on an Agilent 2100 Bioanalyzer. Genomic DNA contamination was assessed by qPCR, using primers for an intronic region of the *MADS AFFECTING FLOWERING 5* (*MAF5*) gene. Only RNA samples with A260/A280 > 2.0, an RNA integrity number (RIN) >7.0, and undetectable gDNA levels, were used for RNA-seq. cDNA was synthesized and sequenced at BGI Genomics (Hong Kong), using a paired-end strategy and a read length of 150 bp, on their proprietary BGISEQ-500 platform.

Clean reads were mapped to the Arabidopsis reference genome (TAIR10) using HISAT2. All subsequent analyses were done in the R programming environment (v.3.6.3; R Core Team, 2020, https://www.R-project.org/; see Supporting Information Methods). Common gene names were retrieved with the biomaRt R package (v.2.47.2) (Drost and Paszkowski, 2017) based on Ensembl gene IDs. Overlaps between different sets of genes were revealed using the Venny online application (http://bioinfogp.cnb.csic.es/tools/venny/index.html). Heat maps for gene expression and metabolite levels were generated using conditional formatting in Excel using maximum and minimum values of Signal log2 ratios for generating the color gradient.

### Statistical analyses

Each data point in the metabolite measurements represents the average value of four to five biological replicates (each being a pool of four to five randomly sampled whole rosettes), with error bars representing the 95% confidence interval. Statistically significant differences were assessed by one-way analysis of variance (ANOVA) Sum of Squares Type II, followed by a Tukey’s post hoc test of honestly significant differences.

Tre6P:sucrose regression plots were built using all biological samples harvested at ED (12 h and 36 h after first dawn) and at EN (24 h and 48 h after first dawn), and used for determining Pearson’s correlation coefficients. Tre6P:sucrose and starch:sucrose ratios were calculated by averaging the ratios obtained for each individual sample at each time point. The results of the Tre6P:sucrose and Starch:sucrose ratios were then subjected to a *t* test for identification of statistical significance between genotypes, or to a one-way ANOVA Sum of Squares Type II, followed by a Tukey’s post hoc test of honestly significant differences, respectively. Rates of starch accumulation and degradation were estimated as described (Moraes et al., 2019), using linear models describing starch levels as a function of time (starch = a.ZT+b).

Statistical analyses were performed using the *R* software (https://www.R-project.org/) and GraphPad Prism version 8.4.0 for Windows (GraphPad Software, La Jolla, CA, USA). In all cases a significance level (α) of 0.05 was used.

### Accession numbers

Sequence data from this manuscript is available in the Arabidopsis Genome Initiative database under these accession numbers: *BGAL4*, At5g56870; *DIN10*, At5g20250; *PYL5*, At5g05440; *TPS1*, At1g78580; *MAF5*, At5g65080; *RZPF34*, At5g22920; *TPS8*, At1g70290; *eIF4A*, At3g13920; *UBC21*, At5g25760; *UBQ10*, At4g05320. Sequence data for otsA is available in GenBank, under the accession number X69160.1. RNAseq data have been deposited in the NCBI GEO database under accession number GSE168382.

## Supplemental data

The following materials are available in the online version of this article.

**Supplemental Figure S1.** Experimental setup for the metabolomic and transcriptomic characterization of SnRK1 mutant lines.

**Supplemental Figure S2.** Impact of SnRK1 on glucose and fructose accumulation.

**Supplemental Figure S3.** Impact of SnRK1 on the sucrose–Tre6P relationship and on *TPS1* transcript accumulation.

**Supplemental Figure S4.** Impact of SnRK1 on starch and starch:sucrose ratios.

**Supplemental Figure S5.** Validation of RNAseq and expression of SnRK1 marker genes.

**Supplemental Table S1.** Metabolite measurements from rosettes of Col-0, *SnRK1α1-OE and sesquiα2* mutants during a 12:12 d-night cycle.

**Supplemental Table S2.** Genes differentially expressed in the *SnRK1α1-OE and sesquiα2* mutants at the ED and EN as compared to the Col-0 control.

**Supplemental Table S3.** Genes differentially expressed in the *SnRK1α1-OE and sesquiα2* mutants both at ED and EN.

**Supplemental Table S4.** Gene ontology enrichment analysis of genes differentially expressed in the *sesquiα2* mutant.

**Supplemental Table S5.** List of primers, antibodies, and plant lines used in this study.

## Supplementary Material

kiab350_Supplementary_Data

## Notes

B.P., J.E.L., M.S., and E.B.G. conceived the research plan and designed the experiments. B.P. and L.M. (TPS1 immunoblots) performed the experiments. TAM assisted with metabolite data analyses. V.M., M.H., and R.F. assisted with sample collection and metabolite profiling. A.G.G.S. and J.L. assisted with RNAseq data analyses. B.P. and E.B.G. analyzed, interpreted data, and wrote the manuscript. J.E.L., M.S., T.A.M., and R.V. interpreted data and edited the manuscript. All the authors proofread the manuscript.

The author responsible for distribution of materials integral to the findings presented in this article in accordance with the policy described in the Instructions for Authors (https://academic.oup.com/plphys/pages/General-Instructions) is: Elena Baena-González (ebaena@igc.gulbenkian.pt).
