# Supplementary material for: Impact of the SnRK1 protein kinase on sucrose homeostasis and the transcriptome during the diel cycle
Source: Plant Physiol. 2021 Jul 28;187(3):1357–73. doi: 10.1093/plphys/kiab350 (PMC8566312; doi:10.1093/plphys/kiab350)
Supplement: kiab350_Supplementary_Data [file kiab350_supplementary_data.zip › Supplemental Information.pdf]

Figure S1

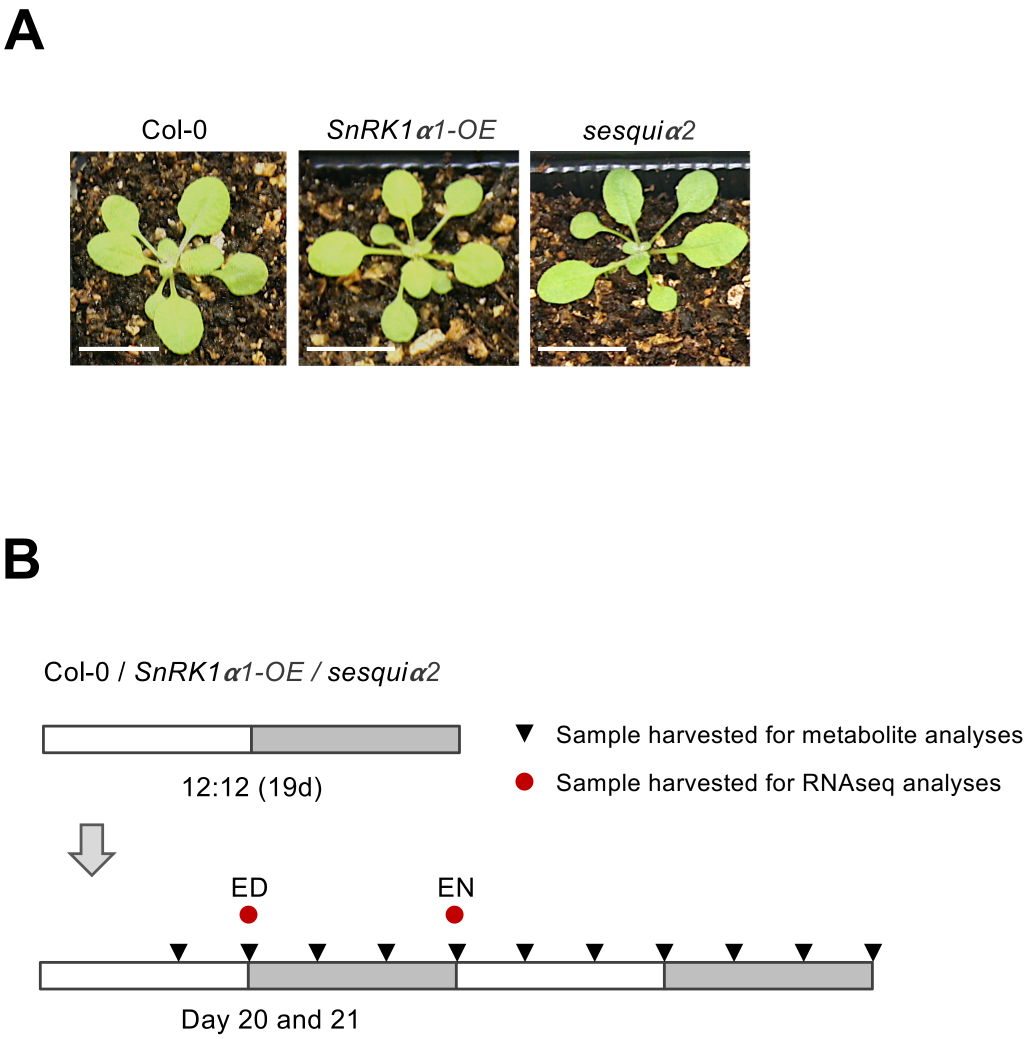

Supplemental Figure S1. Experimental setup for the metabolomic and transcriptomic characterization of SnRK1 mutant lines. A, Representative pictures of the plants used in these analyses are shown [Col-0, *SnRK1α1-OE*, *sesquiα2*]. Scale bar, 1 cm. B, Plants were grown under a 12:12 photoperiod for 19 days. Starting on the 20th day, whole rosettes were harvested every 4 h over a period of 40 h. Black arrows represent the time points used for metabolic measurements, and the red circles represent the end of day (ED) and end of night (EN) time points selected for RNA-seq analyses. The night period is marked in grey.

Figure S2

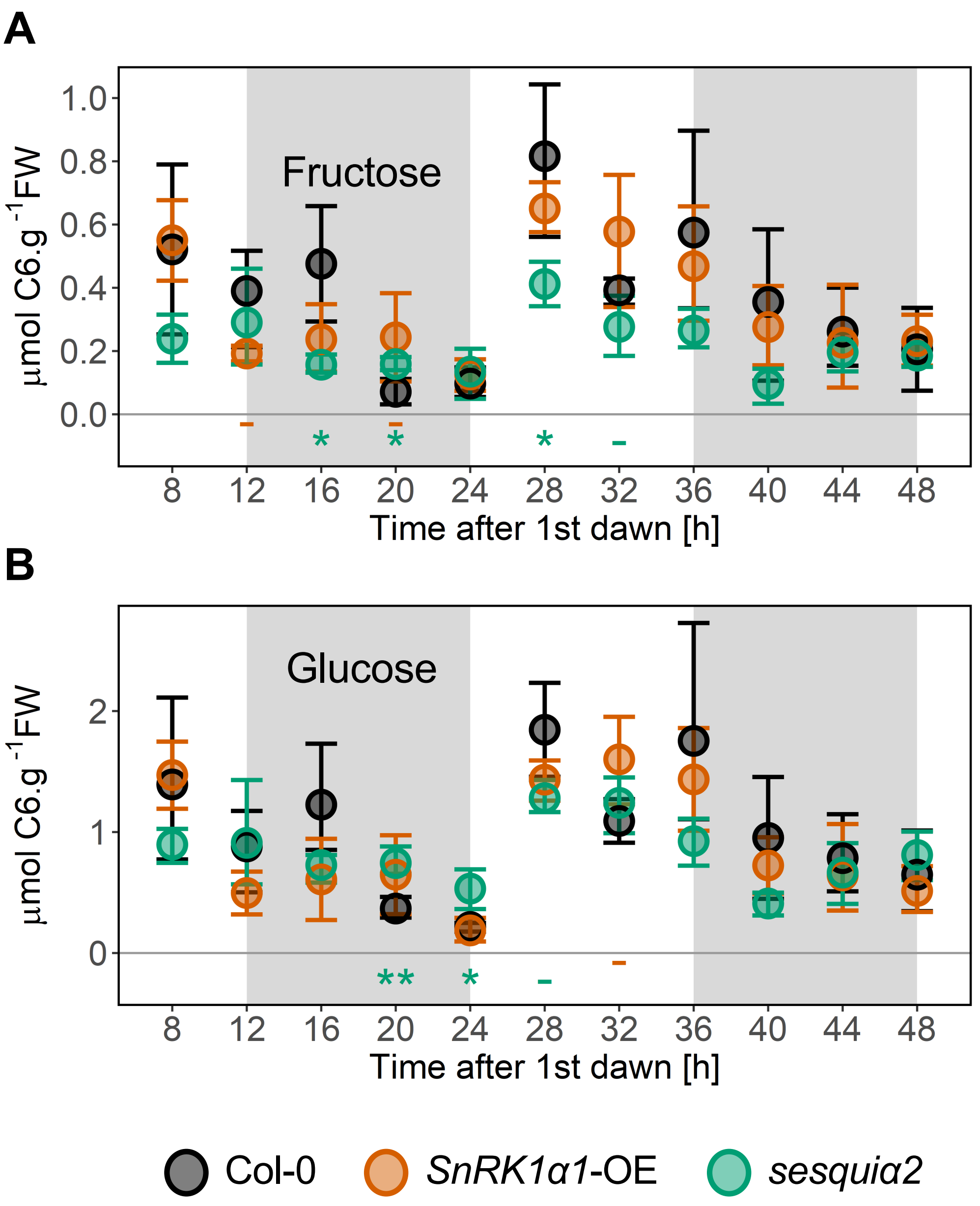

Supplemental Figure S2. Impact of SnRK1 on glucose and fructose accumulation. Fructose (A) and glucose (B) levels were quantified from 20-day old Col-0, *SnRK1α1-OE*, and *sesquiα2* plants grown under a 12:12 photoperiod and harvested every 4 h. The night period is marked in grey. Graphs show the average of 4-5 biological replicates (each composed of a pool of 4-5 randomly sampled whole rosettes) at each time point, with error bars representing the 95% confidence interval. Asterisks denote statistically significant differences tested at each ZT for both genotypes separately (one-way ANOVA with Tukey's post-hoc test of honestly significant differences, HSD). (-), p<0.1 cases in which the Tukey's HSD test resulted in non-significant differences; (*), *p*<0.05; (**), *p*<0.01.

Figure S3

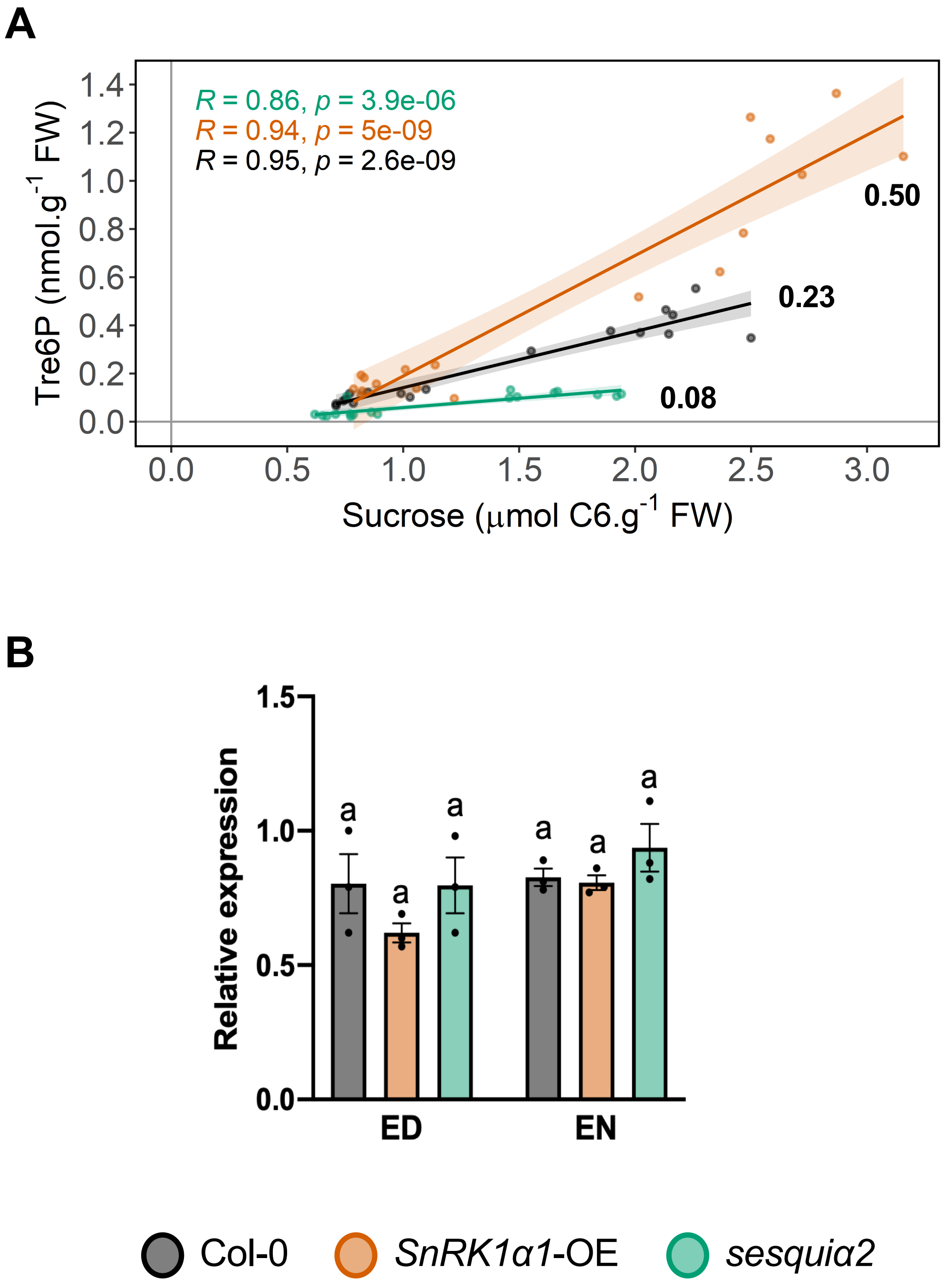

Supplemental Figure S3. Impact of SnRK1 on the sucrose-Tre6P relationship and on *TPS1* transcript accumulation. A, The sucrose content of samples at the end of the day (ED, 12h and 36h after first dawn) and end of the night (EN, 24h and 48h after first dawn) was plotted against the corresponding values of Tre6P. *R*, Pearson correlation coefficient. Black numbers adjacent to each line indicate the slopes of the corresponding sucrose-Tre6P regression. Sucrose levels correspond to hexose equivalents. B, *TPS1* transcript levels quantified by RT-qPCR from 20 d-old Col-0, *SnRK1α1-OE* and *sesquiα2* rosettes at ED and EN. Graph corresponds to the average of 3 independent experiments (error bars, SEM). Letters indicate significantly different groups assessed using the ANOVA and Tukey's HSD post-test. Sucrose levels correspond to hexose equivalents.

Figure S4

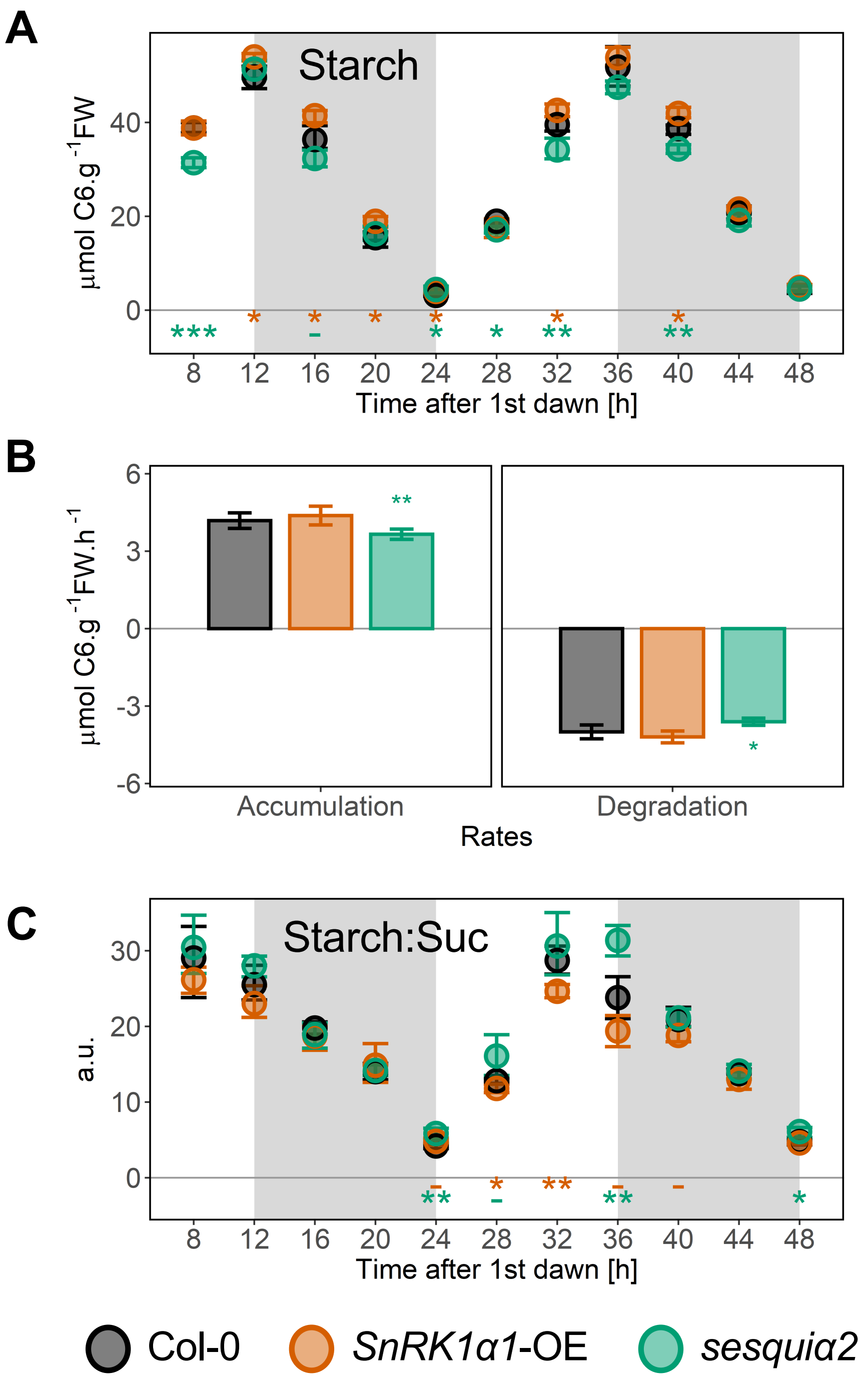

Supplemental Figure S4. Impact of SnRK1 on starch and starch:sucrose ratios. Twenty-day old Col-0, *SnRK1α1-OE*, and *sesquiα2* plants grown under a 12:12 photoperiod were harvested every 4 h for quantifying starch levels (A)**,** rates of starch accumulation during the day (left panel) and starch mobilization during the night (right panel) (B), and starch:sucrose ratios (C). The night period is marked in grey. Time series plots show the average of 4-5 biological replicates (each composed of a pool of 4-5 randomly sampled whole rosettes) at each time point, with error bars representing the 95% confidence interval. Asterisks denote statistically significant differences (one-way ANOVA with Tukey´s HSD post-hoc test). (-), *p*<0.1 cases in which the Tukey's HSD test resulted in non-significant differences; (*), *p*<0.05; (**), *p*<0.01; (***), *p*<0.001. Starch levels correspond to hexose (C6) equivalents. Rates in panel (B) were calculated as the slope of the linear fit of all biological replicates either in the day or night periods. Statistically significant differences to the control Col-0 were tested for both mutants using ANCOVA; *p*-values reported by asterisks as above.

Figure S5

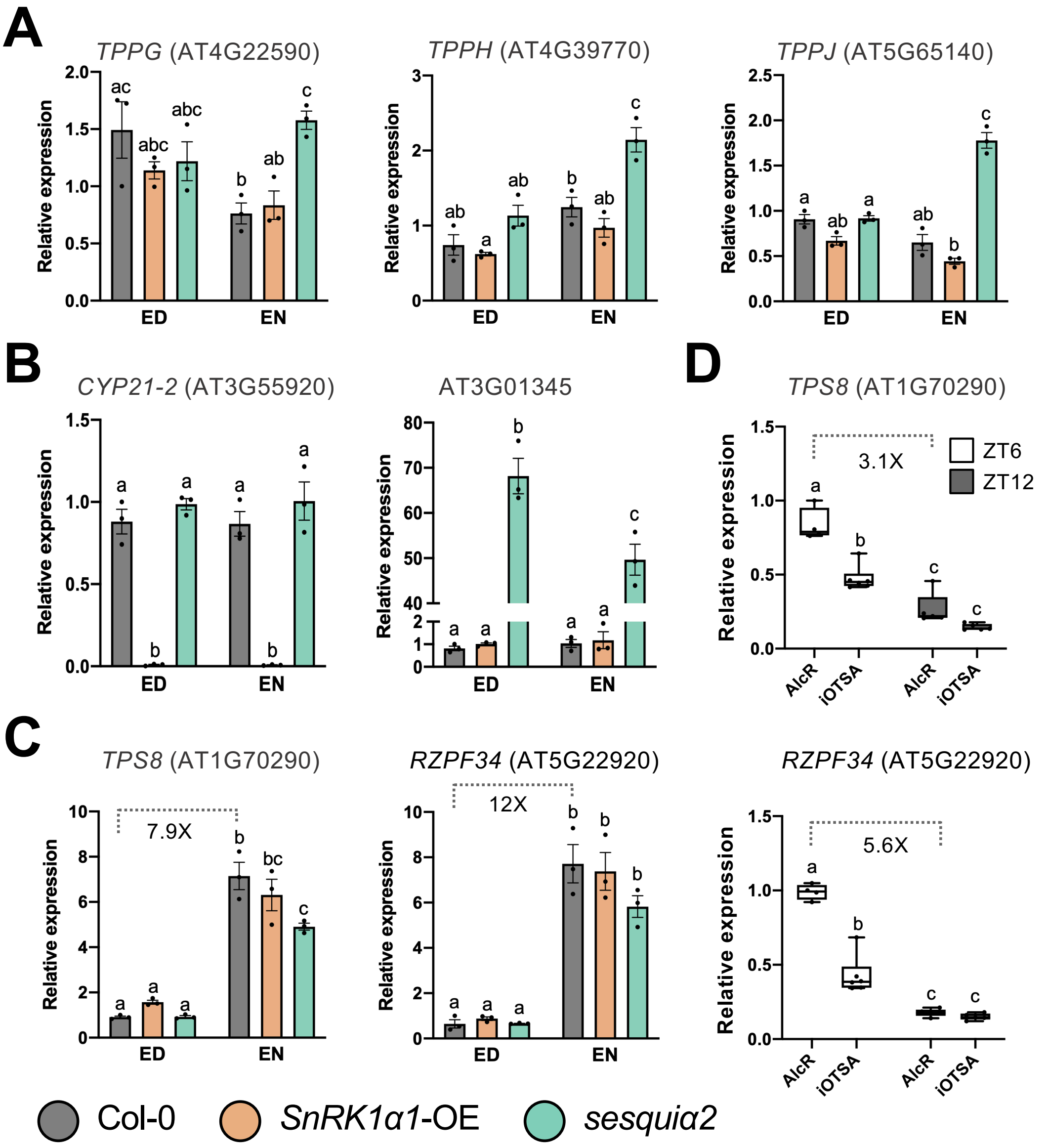

Supplemental Figure S5. Validation of RNAseq and expression of SnRK1 marker genes. RT-qPCR analyses in the indicated genotypes and time points of selected *TPP* genes retrieved as differentially expressed in *sesquiα2* at EN (A), genes affected both at EN and ED in the *SnRK1α1-OE* (left graph) and *sesquiα2* (right graph) RNAseq datasets (B), and two SnRK1 marker genes (Baena-Gonzalez et al., 2007) (C). Graphs correspond to the average of three independent experiments (error bars, SEM). Letters indicate significantly different groups assessed using the ANOVA and Tukey's HSD post-test. D, Induction of OtsA expression in an ethanol-inducible line (iOtsA) and the corresponding empty vector control (AlcR). Ethanol (2% v/v) was sprayed over the plants at ZT0 or at ZT6 and whole rosettes were harvested after 6 h (ZT6 and ZT12 indicate the time of harvest) for RT-qPCR analyses of two SnRK1 marker genes. Quantification of otsA in these samples is shown in Fig. 6a. Values denote expression relative to the ZT6 time point of the AlcR line. Boxplots represent 4-5 biological replicates (each consisting of a pool of three randomly harvested rosettes). Lower and upper box boundaries represent the first and third quantiles, respectively, horizontal lines mark the median and whiskers mark the highest and lowest values. Dots represent individual datapoints. Different letters indicate statistically significant differences (p<0.05, one-way ANOVA with Tukey´s HSD test).

RNA-seq analysis

For RNA-seq analyses, Col-0, *SnRK1α1-OE* and the *sesquia2* mutant were harvested at the end of the day (ED) and end of the night (EN). Three biological replicates (each composed of a pool of 4-5 randomly sampled whole rosettes) were generated from each genotype and time point. Total RNA was extracted from approximately 50 mg of finely ground tissue, with the Qiagen RNeasy® Plant Mini Kit according to the manufacturer's instructions. RNA integrity was confirmed on an Agilent 2100 Bioanalyzer. Genomic DNA contamination was assessed by qRT-PCR, using primers for an intronic region of the *FCL1* gene. Only RNA samples with A260/A280>2.0, an RNA integrity number (RIN)>7.0, and undetectable gDNA levels, were used for RNA-seq. cDNA libraries were synthesized and sequenced at BGI Genomics (Hong-Kong), using a paired-end strategy and a read length of 150 bp, on their proprietary BGISEQ-500 platform.

Clean reads were mapped to the Arabidopsis reference genome (TAIR10) using HISAT2. The resulting table of counts was normalized using a Trimmed Mean of M-values (TMM) procedure (Robinson & Oshlack, 2010) with the *edgeR* R package [v.3.28.1; (Robinson et al., 2009)]. This table was obtained and exported by multiplying the pseudo.counts table by the scaling factors after estimating the scaling factors, the common and the tagwise dispersions with calcNormFactors(), estimateCommonDisp() and estimateTagwiseDisp() functions, respectively. Pairwise comparisons between each SnRK1 mutant line and Col-0 were performed at each time point, using the *edgeR* R package [v.3.28.1; (Robinson *et al.*, 2009)]. Each pairwise comparison was analyzed independently by subsetting the main gene count table to the samples belonging to the groups considered in each comparison. For each pairwise comparison, the subsetted gene count table was imported to edgeR with *DGEList()*, lowly expressed genes filtered out with *filterByExpr()*, with default parameters, with the *min.count=10* and *min.total.count=15* filters being applied across the 3 samples, and the filtered table normalized with the TMM method using the function *calcNormFactors()*. The functions *estimateDisp()* and *glmQLFit()* were used to estimate dispersion and fit the data to a quasi-likelihood negative binomial generalized log-linear model, applying the robust parameter during the estimation of dispersion. Differentially expressed genes were assessed across groups using the *glmQLFTest()* function and a false discovery rate (FDR) cut-off below 0.05 was applied for considering a gene differentially expressed between two conditions. *p*-values were adjusted for multiple comparisons using the Benjamini-Hochberg

correction to control the FDR. Common gene names were retrieved with the biomaRt *R* package (v.2.47.2) (Drost & Paszkowski, 2017) based on Ensembl gene IDs. The plots made in this section were done with the ggplot2 R package (v.3.3.2) (Wickham, 2016). All the analyses described before and made after the mapping step were done in the R programming environment (v.3.6.3; R Core Team, 2020, https://www.R-project.org/).

REFERENCES

Drost HG, Paszkowski J. **2017**. Biomartr: Genomic data retrieval with R. *Bioinformatics* **33**: 1216–1217.

Robinson MD, McCarthy DJ, Smyth GK. **2009**. edgeR: A Bioconductor package for differential expression analysis of digital gene expression data. *Bioinformatics* **26**: 139–140.

Robinson MD, Oshlack A. **2010**. A scaling normalization method for differential expression analysis of RNA-seq data. *Genome Biology* **11**.

Wickham H. **2016**. *ggplot2 Elegant Graphics for Data Analysis (Use R!)*. Springer-Verlag New York. ISBN 978-3-319-24277-4.
